# Up-Regulation of RACGAP1 Promotes Progressions of Hepatocellular Carcinoma Regulated by GABPA via PI3K/AKT Pathway

**DOI:** 10.1155/2022/3034150

**Published:** 2022-08-02

**Authors:** Yang Gu, Baiyang Chen, Deliang Guo, Leyu Pan, Xiaofeng Luo, Jie Tang, Weihua Yang, Yuxian Zhang, Liangqiang Zhang, Jingwen Huang, Rui Duan, Zhigang Wang

**Affiliations:** ^1^Department of Hepatobiliary and Pancreas, The First People's Hospital of Jingmen, Jingmen, Hubei, China; ^2^Department of General Surgery, Xiangyang Central Hospital, Affiliated Hospital of Hubei University of Arts and Science, Xiangyang, Hubei, China; ^3^Department of Hepatobiliary and Pancreas, Zhongnan Hospital of Wuhan University, Wuhan, Hubei, China; ^4^Department of Endocrinology, Suizhou Hospital, Hubei University of Medicine, Hubei, China

## Abstract

Hepatocellular carcinoma (HCC) is one of the dominating tumors causing death due to lack of timely discovery and valid treatment. Abnormal increase of Rac GTPase activating protein 1 (RACGAP1) has been verified to be an oncogene in plenty tumors. The profound mechanism of RACGAP1 was rarely reported in HCC. In this study, we explored the function and mechanism of RACGAP1 in HCC through multiple analysis and experiments. RACGAP1 expression was up-regulated in HCC samples and the high expression of RACGAP1 was an independent prognostic risk factor for HCC patients. Meanwhile, RACGAP1 promoted developments of HCC both in vitro and in vivo. We verified that RACGAP1 promoted proliferation of HCC via PI3K/AKT/CDK2 and PI3K/AKT/GSK3*β*/Cyclin D1 signaling pathway. RACGAP1 accelerated the invasion and metastasis of HCC via phosphorylation of GSK3*β* and nuclear translocation of *β*-catenin. Furthermore, by luciferase reporter assay and Chromatin immunoprecipitation (ChIP) assay, we confirmed Recombinant GA Binding Protein Transcription Factor Alpha (GABPA) regulated the transcription of RACGAP1. All these findings revealed that RACGAP1 promotes the progression of HCC through a novel mechanism, which might be a new therapeutic target for HCC patients.

## 1. Introduction

The high incidence and mortality rates of hepatocellular carcinoma (HCC) caused severe burden worldwide over the past decades [1–3]. HCC is tough to be diagnosed and treated because of its insidious onset and rapid progression. Liver resection, transcatheter arterial chemoembolization (TACE) and liver transplant are the main ways to cure HCC [4–6]. In recent years, the status of immunotherapy and molecular targeted therapy is rapidly rising, which remarkably improved the overall survival and disease-free survival of HCC patients [7, 8]. Thus, it is meaningful to explore the deep pathogenesis and development mechanism of HCC for finding novel vital molecular markers for more efficient treatments.

Abnormal expression of Rac GTPase activating protein 1 (RACGAP1) was involved in the pathogenesis and progression of plenty malignant tumors [9, 10]. Previous research showed that overexpression of RACGAP1 promoted early HCC recurrence by clinical information collection and preliminary molecular experiment [11]. Meanwhile, RACGAP1 was considered as the hub gene related to the prognosis and immune infiltration of HCC according to some data mining studies [12–14]. One study pointed out STAT3 could regulate transcription levels of RACGAP1 leading to overexpression, and RACGAP1 promoted progression of HCC cells by decreasing activation of the Hippo and YAP pathways [15]. However, more novel molecular mechanisms of RACGAP1 in HCC have not been reported yet.

Currently, we indicated that up-regulation of RACGAP1 was existed both in HCC tissues and HCC cells. Meanwhile, HCC patients with higher expression of RACGAP1 had a worse overall survival rate. RACGAP1 was found to facilitate hepatoma cell proliferation and invasion both in vivo and vitro. Notably, we found that RACGAP1 promoted HCC progression via PI3K/AKT pathway, and GABPA transcription factor regulated transcription levels of RACGAP1. In brief, we found RACGAP1 promoted progression of HCC in a novel pathway, which might contribute to a deep understanding of HCC.

## 2. Materials and Methods

### 2.1. Bioinformatics Data

The expression data of RACGAP1 in TCGA-LIHC and GSE40367 was downloaded from UCSC Xena (https://xena.ucsc.edu/public/) and GEO database in NCBI (https://www.ncbi.nlm.nih.gov/pmc/). The overall survival information was also obtained from UCSC Xena. The TFmapper website (http://www.tfmapper.org/), which contains ENCODE (https://www.encodeproject.org/) and GEO database, was used for searching transcription factors of RACGAP1. The data of the correlation of all selected transcription factors with RACGAP1 was came from TCGA-LIHC and CCLE liver tumor cells online database(https://sites.broadinstitute.org/ccle). The binding site regions of transcription factor were predicted by JASPAR online website (http://jaspar.genereg.net/). Our team used KnockTF online website (http://www.licpathway.net/KnockTF/) to predict whether transcription factors have the ability to regulate RACGAP1 transcription (Supplementary File 1 and File 2).

### 2.2. Patient Specimens and Clinical Information Collection

All HCC and adjacent tissues were collected from 70 patients from The First People's Hospital of Jingmen. The pathological results of all these tissues were identified by our own department of pathology according to BCLC Staging System criteria. All tissues were reserved at -80°C with RNA solution after resection of the liver tumor. Patients participated in this study signed informed consent, respectively. The protocols of this study were promised by the ethical committee of The First People's Hospital of Jingmen. All the clinical information and follow-up data were obtained from each patient.

### 2.3. Cell Lines and Culture

In this study, a total number of six human hepatoma cell lines (SMMC7721, HepG2, Hep3B, SK-Hep1, HCCLM3 and Huh7) and the immortalized human hepatic cell line HL-7702 (L02) were obtained from the Cell Bank of Type Culture Collection (CBTCC, the Chinese Academy of Sciences, Shanghai, China). All cell lines were cultured in DMEM (HyClone, USA) supplemented with 10% fetal bovine serum (Gibco, USA) at 37°C containing 5% CO2 in a drippy incubator.

### 2.4. Total RNA Extraction and Quantitative Real-Time PCR

Total RNA was extracted from two kinds of tissues and cells using TRIzol reagent (Invitrogen, USA). Reverse transcription of all mRNAs was conducted by PrimeScript RT reagent Kit with gDNA Eraser (Takara, Tokyo, Japan). Next, quantitative real-time PCR (qRT-PCR) was performed on a CFX Connect Real-time PCR detection system (Bio-Rad, USA) by using a SYBR Green PCR kit (Toyobo, Osaka, Japan). A total 20 *μ*L reaction mixture constituted by 10 *μ*L of 2 × SYBR Mix, 1 *μ*L of forward primer, 1 *μ*L of reverse primer, 6 *μ*L of RNase-free H_2_O and 2 *μ*L of cDNA templates (at a final concentration of 500 *μ*g/*μ*L). The GAPDH was used as an internal control. All expressions were calculated by 2^−*ΔΔ*Ct^ method. All primer sequences used in this were listed in Table S1.

### 2.5. RNA Interference, Plasmid Construction and Cell Transfections

Small interfering RNAs (siRNAs) aiming at knocking down RACGAP1 were designed and compounded by GeneCreat (Wuhan GeneCreate Biological Engineering Co., Ltd, China). The coding sequence of RACGAP1 was loaded into pcDNA3.1 vector for overexpressing RACGAP1. siRNAs and plasmid transfection were operated by Lipofectamin 2000 reagent (Thermo Fisher Scientific, USA) and Lipofectamine 3000 reagent (Thermo Fisher Scientific, USA). All the sequences mentioned above also listed in Table S1.

### 2.6. Immunohistochemistry (IHC) and Immunofluorescence (If)

As for IHC, HCC and non-tumor tissues were fixed by formalin and deparaffinized and rehydrated by xylene and ethanol, respectively. Next, endogenous peroxidase was blocked by 3% H_2_O_2_ for 20 min at room temperature. They were incubated with 10% goat serum for 20 min. Then, we removed the serum and added the RACGAP1 antibody solution overnight at 4°C. Each slice was added 50ul DAKO antibody and incubated at room temperature the next day. Finally, we used hematoxylin for dyeing and alcohol for dehydrating. The expression level of the protein in immunohistochemistry (IHC) were scored according to the extent of cell staining (the percentage of positive cells: no positive cells: 0 score; ≤10%: 1 score; 11~50%: 2 score; 51~80%: 3 score; >80%: 4 score) and the intensity of staining cell (no staining: 0; slight staining: 1; moderate staining: 2; strong staining: 3). Then the score for the extent of cell staining was multiplied by the intensity of staining cell. Score of 0 ~ 3 was negative staining, 4 ~ 6 was weak staining, 7 ~ 9 was moderate staining and 10~12 was strong staining. For immunofluorescence, we used 4% paraformaldehyde to fix and 0.5% Triton X-100 to permeabilize HCC cells. Meanwhile, the primary and secondary antibodies were added in cells according to the steps. After counterstained with DAPI, the results of IF were obtained by a confocal laser-scanning microscope (Olympus, FV3000, Tokyo, Japan).

### 2.7. Cell Proliferation and Colony Assays

We chose Cell Counting Kit-8 (CCK-8, Dojindo, Japan) to measure the capacity of proliferation after transfecting siRNAs and control in Huh7 and HCCLM3 cell lines. Nearly 5 × 10^4^ HCC cells were put into 96-well plates three repeats. Interval of 24 hours, we added 10ul of CCK-8 reagent to 96-well plates and all cells were remained in incubator one hour. Finally, we used the microplate reader to read the absorbance (OD450 nm). The ability of HCC cells for colony was tested by colony formation experiment. Around 8000-1000 cells were seeded in 6-well plates for two weeks. With 4% paraformaldehyde fixation and crystal violet dyeing, all colonies were counted for subsequent statistics.

### 2.8. Cell Migration and Invasion Assays

Wounding healing experiment was used to observe the ability of cell migration. After transfection, we seeded nearly 1 × 10^^6^ HCC cells into 6-well plates without serum. Scratches were operated by a 100ul plastic needle. All plates were cultured at 37°C containing 5% CO2 in a drippy incubator. The wound width was observed and recorded after 24 h with the microscope. As for the ability of invasion, we put around 3 × 10^^4^ HCC cells/well into the upper chambers filled in DMEM without serum. Then, DMEM contained 10% serum was added into under chambers. After incubating for 24 h, we performed the following operations the same as colony assay. Finally, we used an inverted microscope (Olympus Corp, Japan) to observe HCC cell morphology and count the number of cells.

### 2.9. Western Blotting

All tissues and cells were conducted protein extraction with RIPA buffer after transfection 48 h. All protein liquid was stored at -80°C. We put equal amounts of protein on 10% SDS-PAGE for separation and transferred them to PVDF membranes (Millipore, USA). Membranes were soaked in primary antibodies at 4°C overnight after blocked with 5% skimmed milk in TBST for 2 h. All membranes were incubated in second antibodies at room temperature for 2 h by TBST wash three times. We added Clarity Western ECL substrate (Bio-Rad, USA) to the antigen-antibody complex. All antibodies used in this study were listed at Table S1.

### 2.10. Retrospective Experiments

To validate whether RACGAP1 could promote progression of HCC through PI3K/AKT signaling pathway, LY294002, an inhibitor of PI3K/AKT signaling pathway, was diluted with DMSO. And the concentration of LY294002 in DMSO was 10%. The protocols of all retrospective experiments were operated as same as mentioned above.

### 2.11. Experiments In Vivo

All animal experiments were authorized by the Animal Ethics Committee of The First People's Hospital of Jingmen. Four-week-old nude male BALA/c mice were gained from the Animal Center affiliated with the Chinese Academy of Medical Sciences (Beijing, China). We constructed stable HCCLM3 cells with shRNA-NC and shRNA-RACGAP1 transfection and injected them (5 × 10^^5^ cells/mouse) into right armpits of each group mice (6 mice/group). The liver tumor size was measured interval of one week. Two group mice were executed seven weeks later and all tumors were fetched out for comparison. As for lung metastasis assays, approximately 1 × 10^^5^ HCCLM3 cells were injected into tail vein of each mouse. Lungs were fetched for hematoxylin-eosin (H&E) staining eight weeks later.

### 2.12. Chromatin Immunoprecipitation (ChIP) Assay

To validate the regulated relationship of promoter, we conducted Chromatin immunoprecipitation experiment using Magna ChIP-Seq™ Chromatin Immunoprecipitation Kit (Millipore, Billerica, USA) following the corresponding instructions. Firstly, we conducted formaldehyde crosslinking and ultrasonic crushing of cells and impurity removal and antibody feeding. Next, we conducted precipitation and cleaning of immune complexes. All DNA samples were recycled and started PCR operation.

### 2.13. Dual Luciferase Reporter Activity Assay

Based on corresponding protocols of Dual-Luciferase Reporter Assay system (Promega, Madison, USA), Luciferase reporter was used for validating whether the transcription factor has ability to regulate the promoter of RACGAP1. The binding region and corresponding mutant sequences were loaded into the pGL3.0 luciferase reporter vector and transfected with siRNAs into Huh7 cells. Cells were lysed in lysis buffer and detected the luciferase activity after 48 h. Results were showed with normalization.

### 2.14. Statistical Analysis

Three independent experiments are the basic requirements. All statistical analysis were conducted by R (version 4.1.0). Data with two groups was analyzed by t-test. For multiple groups, we used two-way ANOVA analysis. Overall survival information was analyzed by Kaplan-Meier method. Univariate and multivariate regression analysis were analyzed by Cox regression analysis. *P* value <0.05 was considered to be statistically significant.

## 3. Results

### 3.1. RACGAP1 Was up-Regulated in HCC and Leading Poor Overall Survival

**Figure 1:**
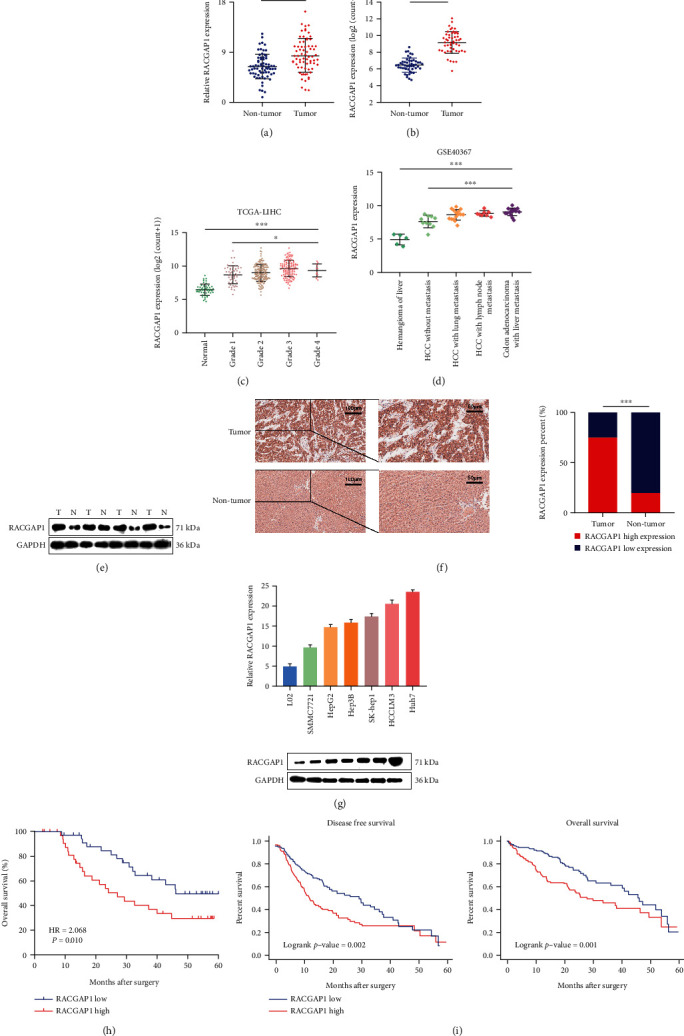
RACGAP1 was up-regulated in HCC and correlated with poor prognosis. (a) Relative expression of RACGAP1 in 70 paired HCC and non-tumor tissues by quantitative RT-PCR. (b) Relative expression of RACGAP1 in 50 paired tissues in TCGA-LIHC. (c) Relative expression of RACGAP1 in different grade stage of HCC in TCGA-LIHC. (d) RACGAP1 expression in metastatic and non-metastatic tissues from GSE40367. (e) The protein levels of RACGAP1 in 4 paired HCC and non-tumor tissues by western-blotting. (f) Immunohistochemistry images of RACGAP1 in HCC and non-tumor tissues (left panel). Semiquantitative data showed RACGAP1 expression analyzed by IHC in HCC tissues compared with non-tumor tissues (right panel). (g) Relative of RACGAP1 in six human hepatoma cell lines (SMMC7721, HepG2, Hep3B, SK-Hep1, HCCLM3 and Huh7) and the immortalized human hepatic cell line HL-7702 (L02). (h) Patients with high expression of RACGAP1 had worse overall survival in 70 HCC patients. (i) Patients with high expression of RACGAP1 had worse disease-free survival and overall survival in TCGA-LIHC. ^∗^p <0.05; ^∗∗∗^p <0.001.

To explore the expression difference of RACGAP1 between HCC and non-tumor tissues, we used 70 paired HCC tissues to conduct RT-PCR. The result manifested that RACGAP1 had higher expression in HCC compared with non-tumor tissues (Figure 1(a)). The TCGA-LIHC 50 paired tissues were also showed the same expression tendency (Figure 1(b)). Meanwhile, high histological grade HCC had higher expression compared with normal tissues (Figure 1(c)). This indicated that higher expression of RACGAP1 might lead worse differentiation of HCC. By searching GSE40367 dataset, we found that RACGAP1 had higher expression in HCC with lung and lymph node metastasis compared with hemangioma and HCC without metastasis (Figure 1(d)). This result implied that high expression of RACGAP1 might had stronger ability leading HCC metastasis. Both western-blotting and IHC demonstrated that RACGAP1 had the same tendency in protein level with the transcriptional level (Figures 1(e) and 1(f)). For subsequent cell validation, RACGAP1 had higher expression in six kinds of HCC cell lines compared with the immortalized liver cell line (L02) by RT-PCR and western-blotting (Figure 1(g)). The clinical information was collected and followed-up. Aberrant expression of RACGAP1 had relationship with histologic grade (p = 0.031), Barcelona Clinic Liver Cancer stage (p = 0.017) and portal vein tumor thrombus (p = 0.041) (Table 1). Moreover, BCLC stage, PVTT and RACGAP1 were independent risk factors for overall survival of HCC patients (Table 2). Lower RACGAP1 expression had longer prognosis in 70 HCC patients (Figure 1(h)). Based on TCGA-LIHC clinical data, both the disease-free survival (p = 0.002) and overall survival (p = 0.001) showed the same tendency (Figure 1(i)). All these results showed that up-regulated RACGAP1 might promote the tumorigenesis and progress of HCC.

### 3.2. RACGAP1 Knockdown Suppressed Proliferation, Invasion and Migration of Hepatoma Cells

**Figure 2:**
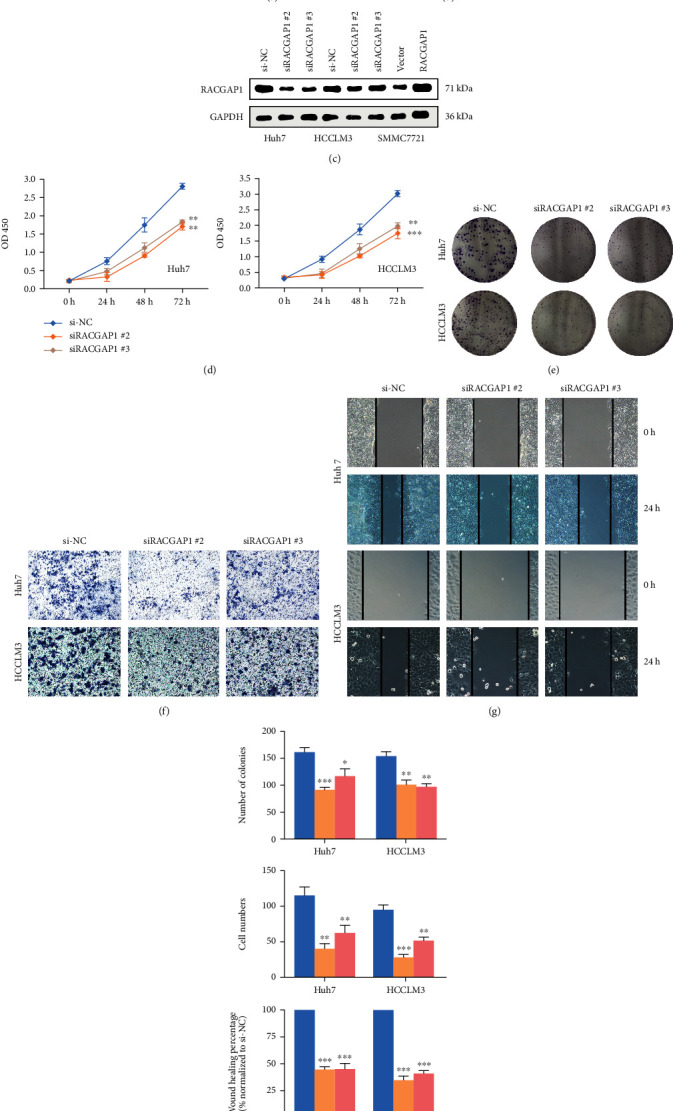
RACGAP1 knockdown inhibited the proliferation, migration and invasion of HCC. The relative expression of RACGAP1 in Huh and HCCLM3 with knockdown (a) and SMMC7721 with over-expression (b) by quantitative RT-PCR. (c) The protein level of RACGAP1 with knockdown and over-expression. CCK-8 (d) and colony formation (e) assays in Huh7 and HCCLM3 with RACGAP knockdown. Invasion (f) and migration (g) tendency in Huh7 and HCCLM3 with RACGAP1 knockdown. (h) The histograms of colony formation, invasion and migration with RACGAP1 knockdown.^∗^p <0.05; ^∗∗^p <0.01; ^∗∗∗^p <0.001.

To explore the underlying function of RACGAP1 in vitro, we chose Huh7 and HCCLM3 for knockdown and SMMC7721 for overexpression according to the result in RT-PCR of hepatoma cells. Three siRNAs (Table S1) were transfected into Huh7 and HCCLM3 cells and the efficiency of knockdown was detected by RT-PCR and western-blotting (Figures 2(a) and 2(c)). Meanwhile, the efficiency of overexpression in SMMC7721 was also measured in same ways (Figures 2(b) and 2(c)). We chose siRACGAP1#2 and siRACGAP1#3 for further experiments based on knockdown results. Previous study emphasized that RACGAP1 could enhance proliferation of cancer cells through Hippo signaling pathway [15]. Our study also demonstrated that knockdown of RACGAP1 reduced the proliferation of hepatoma cells by CCK-8 assay (Figure 2(d)) and colony formation (Figure 2(e)). On the contrary, overexpression of RACGAP1 promoted proliferation of SMMC7721 (Supplementary Figure 1A-1B). To explore the influence of invasion and migration by RACGAP1, both transwell assay and wounding healing assay two vitro experiments suggested that down-regulated RACGAP1 restrained these abilities (Figures 2(f) and 2(h)). Meanwhile, the abilities of invasion and migration were ameliorated after RACGAP1 overexpression in SMMC7721 (Supplementary Figure 1C-1D). In brief, down-regulation of RACGAP1 reduced proliferation, invasion and migration in vitro and up-regulation of RACGAP1 had the opposite tendency.

### 3.3. Knockdown of RACGAP1 Attenuated Tumor Growth and Metastasis In Vivo

**Figure 3:**
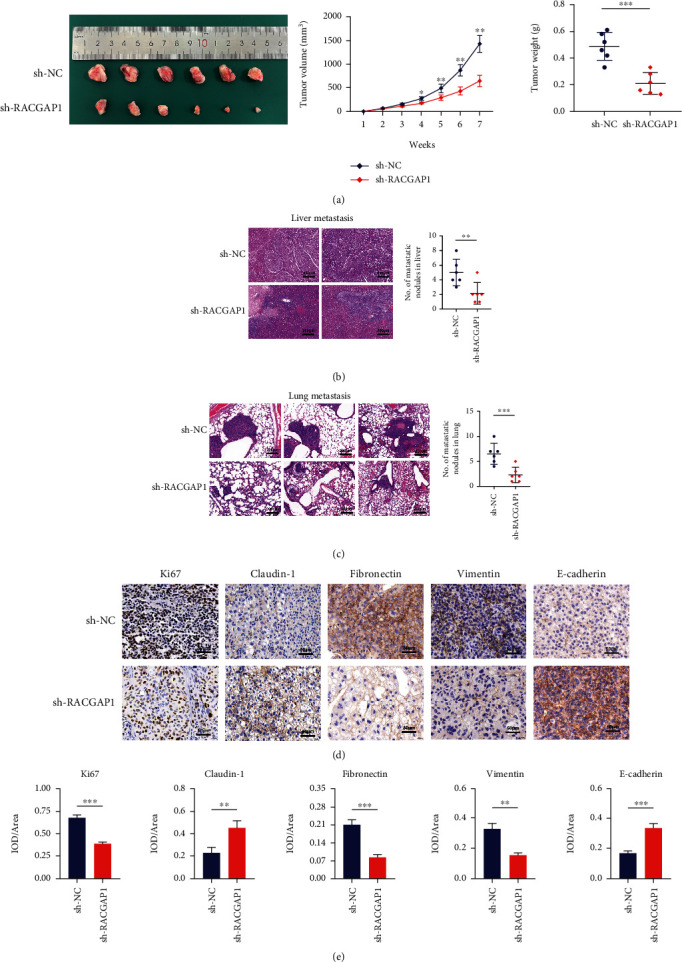
RACGAP1 knockdown suppressed HCC growth and metastasis in vivo. (a) Representative images of tumors removed from mice (left panel); Tumor growth curves (middle panel) and tumor weights (right panel) at different time points in vivo. (b) Representative images of H&E staining of intrahepatic metastasis. (c) Representative images of H&E staining of pulmonary metastasis. (d) Immunohistochemistry and Semiquantitative data (e) revealed that the expression of Ki67, Fibronectin and vimentin was declined, while the expression of claudin-1 and E-cadherin was up-regulated in the sh-RACGAP1 HCCLM3 xenograft tumors. ^∗^p <0.05; ^∗∗^p <0.01; ^∗∗∗^p <0.001.

Knockdown of RACGAP1 suppressed proliferation, invasion and migration of hepatoma cells in our preliminary study. However, the function of RACGAP1 remains unknown in vivo. Nude male BALA/c mice were injected HCCLM3 cells stably transfected with sh-RACGAP1 and control cells. Compared with sh-NC group, tumor size and weight were obviously diminished in sh-RACGAP1 (Figure 3(a)). Meanwhile, down-regulation of RACGAP1 could suppressed tumor growth through the tumor growth curve (Figure 3(a)). These results indicated that RACGAP1 had influence on tumorigenesis of HCC. Besides, two models were constructed to investigate the function of RACGAP1 for metastasis in vivo. The number and size of intrahepatic metastasis and lung metastasis were distinctly declined with hematoxylin-eosin staining (Figures 3(b) and 3(c)). The IHC staining results demonstrated that some markers of proliferation and metastasis were changed. Down-regulation of RACGAP1 reduced Ki67, Fibronectin and Vimentin (Figures 3(d) and 3(e)). The expression of Claudin-1 and E-cadherin were enhanced after reducing RACGAP1 (Figures 3(d) and 3(e)). All these findings revealed that increased RACGAP1 could promote the proliferation and metastasis of HCC.

### 3.4. RACGAP1 Promoted Proliferation and Invasion via PI3K/AKT Pathway

**Figure 4:**
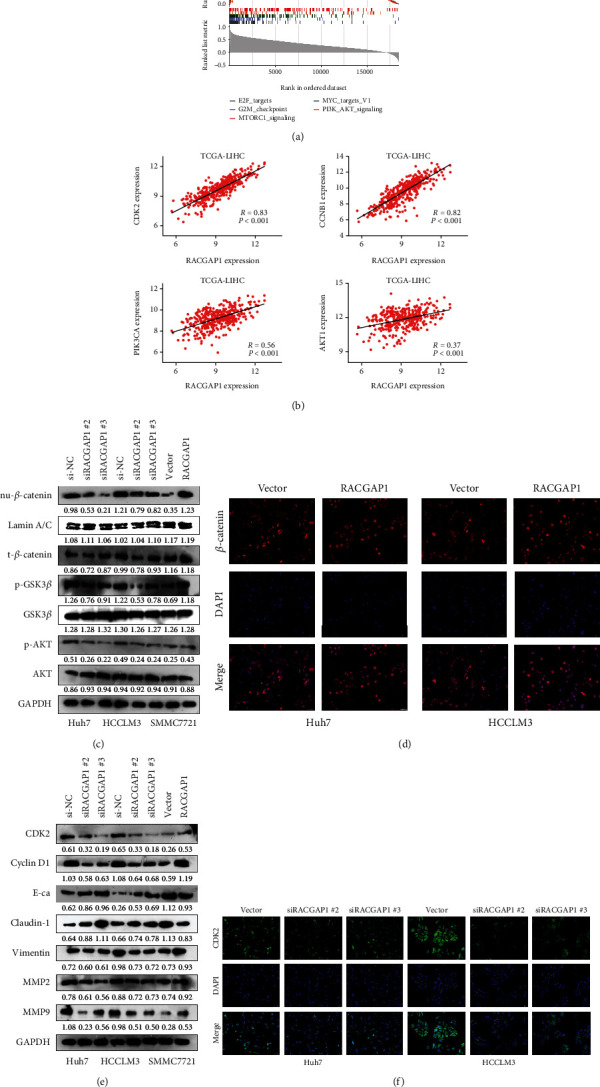
RACGAP1 promoted proliferation and metastasis of HCC through PI3K/AKT signaling pathway. (a) The underlying pathways of RACGAP1 participation analyzed by GSEA (Gene Set Enrichment Analysis). (b) The proliferation markers (CDK2 and CCNB1) and key genes (PIK3CA and AKT1) in PI3K/AKT signaling pathway showed high correlation with RACGAP1 in TCGA-LIHC. (c) The nuclear *β*-catenin (nu-*β*-catenin), p-GSK3*β* and p-AKT were obviously diminished in Huh7 and HCCLM3 with si-RACGAP1, while increased in SMMC7721 with overexpression of RACGAP1. (d) RACGAP1 overexpression promoted *β*-catenin nuclear translocation by immunofluorescence. (e) The protein levels of proliferation markers (CDK2 and Cyclin D1), EMT markers (E-ca, Claudin-1 and Vimentin) and MMPs (MMP2 and MMP9) in HCC cell lines with RACGAP1 knockdown and overexpression. (f) RACGAP1 knockdown suppressed CDK2 expression in Huh7 and HCCLM3 cell lines.

**Figure 5:**
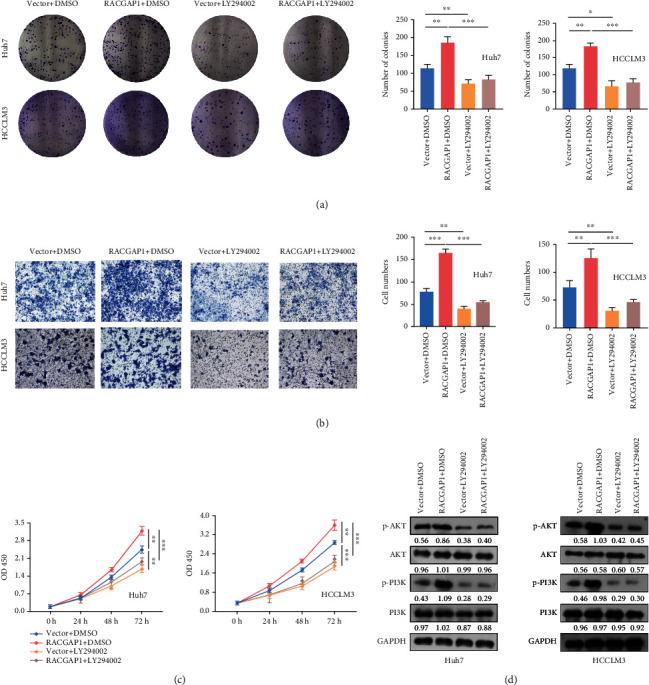
LY294402 blocked the function of RACGAP1. Clone formation (a), transwell (b) and CCK-8 (c) after cotransfection with vector or RACGAP1 and DMSO or LY294002 in Huh7 and HCCLM3 cell lines. (d) The expression of core markers in PI3K/AKT signaling pathway after cotransfection with vector or RACGAP1 and DMSO or LY294002 by western-blotting in Huh7 and HCCLM3 cell lines. ^∗^p <0.05; ^∗∗^p <0.01; ^∗∗∗^p <0.001.

We performed Gene Set Enrichment Analysis (GSEA) to explore deep mechanism why up-regulation of RACGAP1 could promote the proliferation and metastasis in vitro and vivo. Five main signaling pathways were enriched, including E2F_targets, G2M_checkpoint, MTORC1_signaling pathway, MYC_targets and PI3K/AKT signaling pathway (Figure 4(a)). Plenty researches have showed activation of PI3K/AKT signaling pathway promoted proliferation, invasion and metastasis in HCC [16, 17]. The transcription level of MYC and RACGAP1 showed no correlation. The correlation of key genes PIK3CA and AKT1 in PI3K/AKT signaling pathway with RACGAP1 were 0.56 and 0.37, respectively (Figure 4(b)). Two key genes, CDK2 and CCNB1, which had strong influence on proliferation had high correlation with RACGAP1 (Figure 4(b)). All these results implied that RACGAP1 might promote proliferation and invasion through PI3K/AKT pathway. The epithelial-to-mesenchymal transition (EMT) has high correlation with tumor invasion and metastasis in HCC [18]. Studies emphasized that phosphorylation of GSK3*β*, regulated by AKT, restrained the expression of *β*-catenin [19]. Our study showed the levels of phosphorylation of AKT (p-AKT) and GSK3*β* (p- GSK3*β*) was obviously decreased with transfection of RACGAP1 siRNAs in Huh7 and HCCLM3 cell lines (Figure 4(c)). The nuclear *β*-catenin (nu-*β*-catenin) was also reduced (Figure 4(c)). Meanwhile, over-expressed RACGAP1 had the opposite results. CDK2 and Cyclin D1 were the downstream molecules of AKT and GSK3*β*, respectively [20]. The protein levels of proliferation, CDK2 and Cyclin D1, were diminished with siRACGAP1 in Huh7 and HCCLM3 cell lines (Figure 4(e)). The tendency was inverse with over-expressed RACGAP1 in SMMC7721 cell line (Figure 4(e)). PI3K/AKT signaling pathway influenced EMT in multiple ways [21]. With down-regulation of RACGAP1, E-ca and Claudin-1 were enhanced and Vimentin protein was declined. These three EMT markers had inverse tendency with over-expression of RACGAP1 (Figure 4(e)). Meanwhile, two matrix metalloproteinases (MMP2 and MMP9) related to metastasis were decreased with siRACGAP1 (Figure 4(e)). Immunofluorescence showed that the translocation of *β*-catenin was increased with over-expression of RACGAP1 in Huh7 and HCCLM3 cell lines (Figure 4(d)). Meanwhile, we found the protein levels of CDK2 were reduced after knockdown of RACGAP1 by Immunofluorescence (Figure 4(f)). To further validate whether RACGAP1 could promote proliferation and invasion via PI3K/AKT pathway. LY294002 [22], an inhibitor of PI3K/AKT signaling pathway, was used for retrospective experiment. The number of colonies was reduced with RACGAP1 overexpression by adding LY294002 (Figure 5(a)). Meanwhile, the invasion ability induced by overexpression of RACGAP1 was blocked after intercepting PI3K/AKT signaling pathway (Figure 5(b)). The condition of proliferation had the same tendency in Huh7 and HCCLM3 cell lines (Figure 5(c)). The core markers of PI3K/AKT signaling pathway were also repressed with overexpression of RACGAP1 by using LY294002 (Figure 5(d)). Taken together, these retrospective experiments suggested that RACGAP1 promoted proliferation and invasion via PI3K/AKT pathway.

### 3.5. Up-Regulation of GABPA Enhance Expression of RACGAP1 in Hepatoma Cells

**Figure 6:**
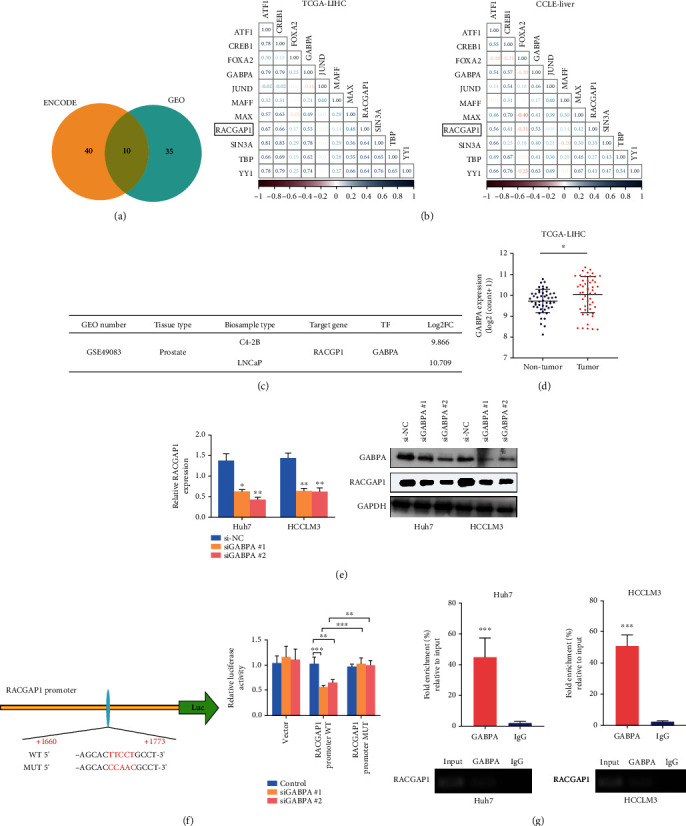
Excessive activation of GABPA promoted RACGAP1 transcription in HCC. (a) Total ten transcription factors (TFs) were intersected from ENCODE and GEO database by Veen. (b) The correlations of ten TFs with RACGAP1 in TCGA-LIHC and CCLE-Liver. (c) RACGAP1 was obviously changed with knockdown of GABPA in prostate tissues in GSE49083. (d) GABPA had higher expression in HCC compared with non-tumor tissues. (e) The efficiency of GABPA knockdown in Huh7 and HCCLM3 cell lines. (f) Schematic diagram of GABPA binding site on RACGAP1 promoter and the mutant RACGAP1 promoter (left panel); Inactivation of GABPA obviously reduced wild type but not mutant RACGAP1 promoter luciferase activity (right panel). (g) Chromatin immunoprecipitation (ChIP) assay showed GABPA binding to the promoter of RACGAP1 in Huh7 and HCCLM3 cells.^∗^p <0.05; ^∗∗^p <0.01; ^∗∗∗^p <0.001.

It has been reported that E2F3, one transcription factor (TF) of E2F family proteins, regulated expression of RACGAP1 in in esophageal squamous cell carcinoma. Transcriptions of mRNAs are complicated which means one mRNA might have various transcription factors. The situation of transcription of RACGAP1 in HCC has not been reported yet. A total number of ten TFs were screened by ENCODE and GEO website after intersection (Figure 6(a)). The correlation of eleven mRNAs, including ten TFs and RACGAP1, were calculated using TCGA-LIHC and CCLE-Liver (Figure 6(b)). Correlations of majority TFs (MAX, YY1, ATF1, CREB1, SIN3A and GABPA) were more than 0.3. By searching TF-knockdown online website, our team found the expression of RACGAP1 was obviously affected after knock-down of GABPA in C4-2B and LNCaP cells (Figure 6(c)). To validate whether GABPA regulated RACGAP1, we acquired siGABPA #1 and #2 for experiments in vitro. The expression of RACGAP1 was down-regulated significantly both in RT-PCR and Wester-blotting (Figure 6(e)) after knockdown GABPA. The expression of GABPA was higher in HCC compared with non-tumor tissues in TCGA-LIHC (Figure 6(d)). Next, the JASPAR online website was used to explore the binding region of GABPA and promoter of RACGAP1. The result of luciferase demonstrated that the 5'UTR site (+1660 to +1773 from start site of transcription) was combined by GABPA (Figure 6(f)). Meanwhile, ChIP-PCR experiment was also conducted to validate this result in Huh7 and HCCLM3 cells (Figure 6(g)). All these findings revealed that overexpression of GABPA accelerated up-regulation of RACGAP1 in HCC.

## 4. Discussion

Studies about RACGAP1 have been reported in the last decade. One recent research emphasized that Radiotherapy, frequently used to cancer treatment, might reduce tumor cell activity and restrained capacity of invasion and metastasis by down-regulation of RACGAP1 [23]. Aberrant expression of RACGAP1 caused tumorigenesis and tumor progression in multiple tumors [10, 24, 25]. Specific and novel molecular mechanisms pressingly need to be discovered.

In this study, we proved that RACGAP1 was over-expression both in HCC tissues and hepatoma cell lines. Up-regulation of RACGAP1 had relationship with histologic grade, BCLC stage and PVTT in HCC by clinical information analysis. Meanwhile, patients with high expression of RACGAP1 had less overall survival rate and high expression of RACGAP1 was an independent prognostic risk factor, which is consistent with previous study [26]. Our team provided and demonstrated a novel theory that RACGAP1 regulated by GABPA could promote proliferation and invasion in HCC via PI3K/AKT pathway both in vitro and vivo.

PI3K/AKT signaling pathway plays an important role in information transfer and signal transduction in tumors [27, 28]. One study indicated that Ras and Rho family small GTPases could bind PI3K directly and reinforce PI3K activity [29]. According to the signaling pathway map, AKT could activate CDK2 directly and CDK2 finishes G1/S transition in cell cycle [30]. And another vital cell cycle protein Cyclin D1, regulated by GSK3*β* is suppressed by AKT [31]. These two proteins could regulate proliferation in HCC, which also had been verified in our study. The stability and nuclear translocation of the intracellular *β*-catenin influence Wnt signaling pathway directly and indirectly. GSK3*β* played a vital role both in these two sides [32]. The phosphorylation of GSK3*β* phosphorylates *β*-catenin and lead to degradation of *β*-catenin [33]. Meanwhile, *β*-catenin is transferred into nucleus with down-regulation of E-cadherin, which results in EMT in HCC [34]. Our study also revealed that up-regulation of RACGAP1 could enhance nuclear translocation of *β*-catenin, which leaded invasion and metastasis in HCC. Interestingly, MMP-2 and MMP-9, two main matrix metalloproteinases in decomposing collagen include type IV and I were also influenced by RACGAP1. This result might reveal RACGAP1 promoted metastasis through extra cellular matrix (ECM). GABPA, one of the ETS family transcription factors, is recommended as an oncogenic protein in previous study [35]. Meanwhile, some researchers considered that GABPA might act as a tumor suppressor and restrained invasion and metastasis in thyroid carcinoma [36]. One study indicated that abnormal expression of GABPA has relationship with progression of HCC and this transcription factor acted as a tumor suppressor [37]. We insisted that GABPA, acted as an oncogenic protein, promoted transcription of RACGAP1 in our study.

**Figure 7:**
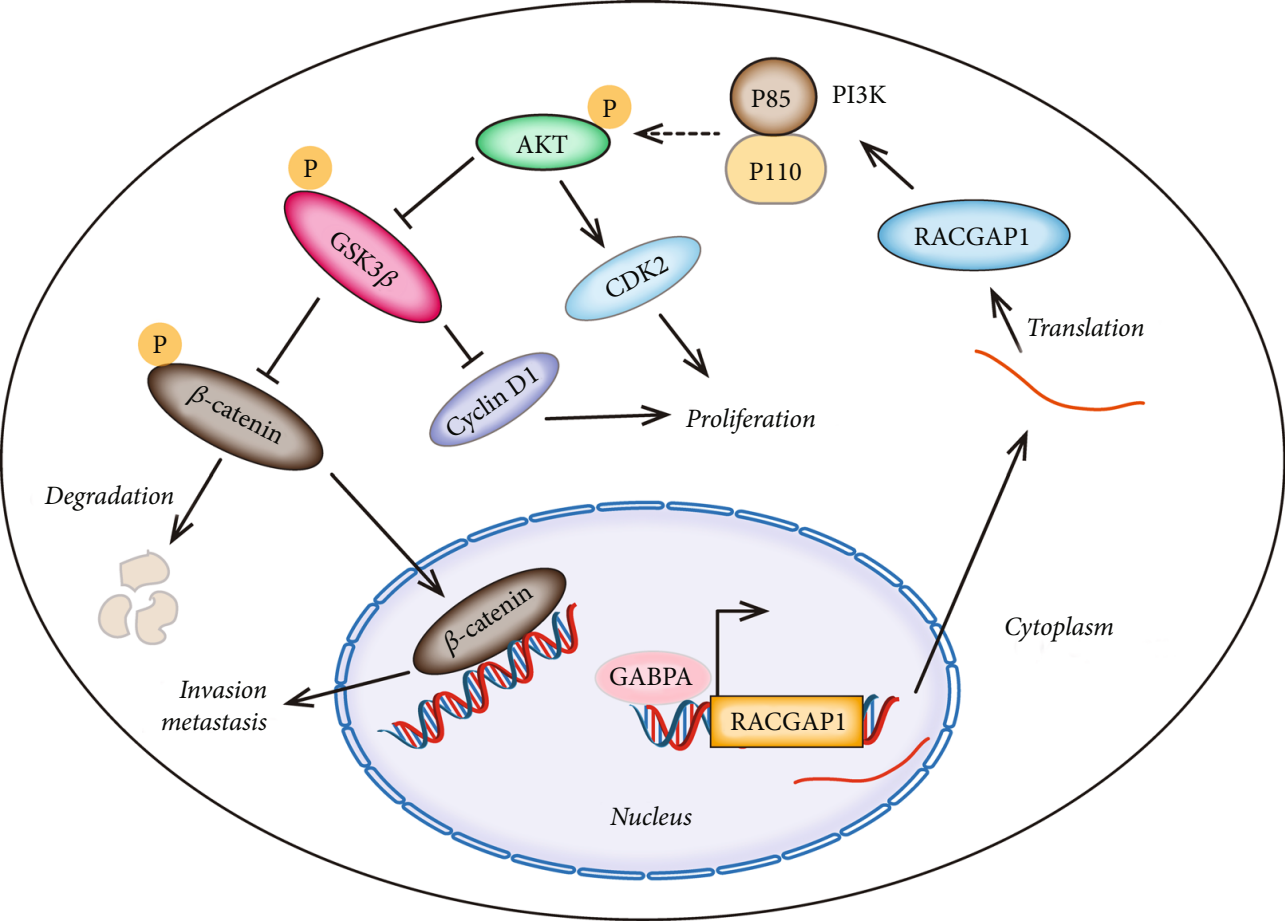
Schematic diagram shows that RACGAP1 promotes proliferation via AKT/CDK2 and GSK3*β*/Cyclin D1 and metastasis via GSK3*β*/*β*-catenin. And GABPA could directly regulate the transcription of RACGAP1.

In summary, our findings confirmed that RACGAP1 acted as an oncogene consistent with previous studies. RACGAP1 facilitated proliferation and invasion via PI3K/AKT signaling pathway both in vitro and vivo. Meticulously speaking, RACGAP1 promoted proliferation of HCC via PI3K/AKT/CDK2 and PI3K/AKT/GSK3*β*/Cyclin D1 signaling pathway. RACGAP1 accelerated invasion and metastasis of HCC via phosphorylation of GSK3*β* and nuclear translocation of *β*-catenin. And GABPA, binding the promoter region of RACGAP1, promoted its transcription (Figure 7). All these findings revealed a novel molecular mechanism which might contribute to diagnosis and treatments of HCC patients.

## Figures and Tables

**Table 1:** The relationship between RACGAP1 and clinical characteristics of HCC patients. BCLC: Barcelona Clinic Liver Cancer; PVTT: Portal Vein Tumor Thrombus.

Characteristics	Number of cases	RACGAP1 expression		P value
		High(n =35)	Low(n =35)	
*Gender*				0.274
Female	18	11	7	
Male	52	24	28	
*Age(years)*				0.811
<65	33	16	17	
≥65	37	19	18	
*Tumor size(cm)*				0.068
<5	49	21	28	
≥5	21	14	7	
*HBV infection*				0.274
No	18	7	11	
Yes	52	28	24	
*Multi-noodular*				0.124
No	57	26	31	
Yes	13	9	4	
*Serum AFP(μg/L)*				0.138
<400	44	19	25	
≥400	26	16	10	
*Cirrhosis*				0.794
No	21	10	11	
Yes	49	25	24	
*Histologic grade*				**0.031**∗
Well or moderate	57	25	32	
Poor	13	10	3	
*BCLC stage*				**0.017**∗
A	56	24	32	
B + C	14	11	3	
*PVTT*				**0.041**∗
No	55	24	31	
Yes	15	11	4	

**Table 2:** Univariate and multivariate Cox analysis for overall survival of RACGAP1. BCLC: Barcelona Clinic Liver Cancer; PVTT: Portal Vein Tumor Thrombus.

Characteristics	Univariate analysis			Multivariate analysis		
	Hazard ratio	95% CI	P value	Hazard ratio	95% CI	P value
Gender	0.963	0.564-1.643	0.889			
Age(years)	1.183	0.743-1.885	0.478			
Tumor size(cm)	1.350	0.840-2.169	0.215			
HBV infection	1.351	0.772-2.364	0.293			
Multi-noodular	0.866	0.465-1.613	0.651			
Serum AFP(*μ*g/L)	1.248	0.783-1.988	0.351			
Cirrhosis	0.943	0.578-1.541	0.816			
Histologic grade	2.822	1.603-4.966	**<0.001**	1.773	0.924-3.401	0.085
BCLC stage	2.337	1.383-3.949	**0.002**	2.172	1.248-3.780	**0.006**
PVTT	3.089	1.836-5.199	**<0.001**	2.159	1.164-4.006	**0.015**
RACGAP1 expression	1.251	1.129-1.387	**<0.001**	1.140	1.021-1.273	**0.020**

## Data Availability

The authors declare that all data and materials are available on request.

## Supplementary Materials

Supplementary Materials Supplementary Figure 1: Over-expression of RACGAP1 promotes HCC growth (A and B) and metastasis (C and D) in SMMC7721. ^∗∗^p <0.01; ^∗∗∗^p <0.001. Table S1: The sequences of all primers and the information of all antibodies in our study. Supplementary File: The results of GEO (File 1) and ENCODE (File 2) for transcription factors prediction of RACGAP1.
