# Supplementary material for: Up-Regulation of RACGAP1 Promotes Progressions of Hepatocellular Carcinoma Regulated by GABPA via PI3K/AKT Pathway
Source: Oxid Med Cell Longev. 2022 Aug 2;2022:3034150. doi: 10.1155/2022/3034150 (PMC9363186; doi:10.1155/2022/3034150)
Supplement: Supplementary Materials — Supplementary Figure 1: Over-expression of RACGAP1 promotes HCC growth (A and B) and metastasis (C and D) in SMMC7721. ∗∗p <0.01; ∗∗∗p <0.001. Table S1: The sequences of all primers and the information of all antibodies in our study. Supplementary File: The results of GEO (File 1) and ENCODE (File 2) for transcription factors prediction of RACGAP1. [file 3034150.f1.zip › Supplementary File1.docx]

|  | sampleID | feature_type | start | end | score | dis | symbol | attri | p | q | chrom | GSM | bs1 | bs2 | bs3 | IP | species | type | source_name_ch1 |
| --- | --- | --- | --- | --- | --- | --- | --- | --- | --- | --- | --- | --- | --- | --- | --- | --- | --- | --- | --- |
| 1 | 45296 | 1 | 50025748 | 50026029 | 6.118 | -331 | RACGAP1 | promoter-TSS (NM_001320003) | 13.41939 | 9.52784 | chr12 | GSM733774 | HepG2 | Epithelium | Liver | H2AZ | human | TF | HepG2 |
| 70 | 8108 | 1 | 50025758 | 50025904 | 5.60196 | -274 | RACGAP1 | promoter-TSS (NM_001320003) | 8.16067 | 5.2442 | chr12 | GSM748543 | HepG2 | Epithelium | Liver | POLR2A | human | TF | HepG2_PolII |
| 48 | 46256 | 1 | 50025645 | 50025940 | 10.34602 | -235 | RACGAP1 | promoter-TSS (NM_001320003) | 39.39763 | 36.48125 | chr12 | GSM803368 | HepG2 | Epithelium | Liver | POLR2A | human | TF | HepG2 |
| 41 | 46247 | 1 | 50025647 | 50025927 | 15.66652 | -230 | RACGAP1 | promoter-TSS (NM_001320003) | 106.8083 | 102.9418 | chr12 | GSM1010821 | HepG2 | Epithelium | Liver | POLR2A | human | TF | HepG2 |
| 67 | 7341 | 1 | 50025642 | 50025900 | 12.57545 | -214 | RACGAP1 | promoter-TSS (NM_001320003) | 59.33376 | 56.1206 | chr12 | GSM803448 | HepG2 | Epithelium | Liver | HEY1 | human | TF | HepG2 |
| 33 | 46238 | 1 | 50025571 | 50025942 | 4.97749 | -199 | RACGAP1 | promoter-TSS (NM_001320003) | 11.06199 | 8.20696 | chr12 | GSM1010875 | HepG2 | Epithelium | Liver | TEAD4 | human | TF | HepG2 |
| 34 | 46239 | 1 | 50025630 | 50025847 | 3.94048 | -181 | RACGAP1 | promoter-TSS (NM_001320003) | 7.0567 | 4.52121 | chr12 | GSM803404 | HepG2 | Epithelium | Liver | HNF4G | human | TF | HepG2 |
| 22 | 46224 | 1 | 50025636 | 50025804 | 4.17836 | -163 | RACGAP1 | promoter-TSS (NM_001320003) | 6.69163 | 3.97282 | chr12 | GSM1010831 | HepG2 | Epithelium | Liver | ZBTB7A | human | TF | HepG2 |
| 25 | 46227 | 1 | 50025531 | 50025856 | 13.71768 | -136 | RACGAP1 | promoter-TSS (NM_001320003) | 61.099 | 58.0103 | chr12 | GSM803403 | HepG2 | Epithelium | Liver | FOXA2 | human | TF | HepG2 |
| 66 | 7323 | 1 | 50025511 | 50025852 | 13.82623 | -124 | RACGAP1 | promoter-TSS (NM_001320003) | 63.58158 | 60.57455 | chr12 | GSM803461 | HepG2 | Epithelium | Liver | FOXA1 | human | TF | HepG2 |
| 6 | 45798 | 1 | 50025480 | 50025867 | 5.98205 | -116 | RACGAP1 | promoter-TSS (NM_001320003) | 15.99684 | 12.33685 | chr12 | GSM935579 | HepG2 | Epithelium | Liver | RCOR1 | human | TF | HepG2 |
| 13 | 45815 | 1 | 50025493 | 50025850 | 5.6984 | -114 | RACGAP1 | promoter-TSS (NM_001320003) | 13.3554 | 11.04684 | chr12 | GSM935305 | HepG2 | Epithelium | Liver | MAFK | human | TF | HepG2 |
| 52 | 56042 | 1 | 50025412 | 50025931 | 22.09508 | -114 | RACGAP1 | promoter-TSS (NM_001320003) | 100.1781 | 96.23115 | chr12 | GSM1861924 | HepG2 | Epithelium | Liver | GABPA | human | TF | HepG2 cells |
| 65 | 7322 | 1 | 50025513 | 50025822 | 14.63461 | -110 | RACGAP1 | promoter-TSS (NM_001320003) | 54.50911 | 51.42736 | chr12 | GSM803432 | HepG2 | Epithelium | Liver | FOXA1 | human | TF | HepG2 |
| 44 | 46251 | 1 | 50025384 | 50025932 | 4.53322 | -101 | RACGAP1 | promoter-TSS (NM_001320003) | 12.92726 | 9.74731 | chr12 | GSM1010740 | HepG2 | Epithelium | Liver | MBD4 | human | TF | HepG2 |
| 47 | 46255 | 1 | 50025440 | 50025853 | 15.89276 | -89 | RACGAP1 | promoter-TSS (NM_001320003) | 81.57071 | 76.94944 | chr12 | GSM803418 | HepG2 | Epithelium | Liver | ZBTB33 | human | TF | HepG2 |
| 55 | 56045 | 1 | 50025348 | 50025931 | 23.09088 | -82 | RACGAP1 | promoter-TSS (NM_001320003) | 250.5505 | 245.8174 | chr12 | GSM1861927 | HepG2 | Epithelium | Liver | GABPA | human | TF | HepG2 cells |
| 2 | 45788 | 1 | 50025561 | 50025713 | 5.21866 | -80 | RACGAP1 | promoter-TSS (NM_001320003) | 10.51067 | 7.51284 | chr12 | GSM935335 | HepG2 | Epithelium | Liver | MAZ | human | TF | HepG2 |
| 45 | 46253 | 1 | 50025485 | 50025784 | 4.65806 | -77 | RACGAP1 | promoter-TSS (NM_001320003) | 10.03196 | 7.18441 | chr12 | GSM1010784 | HepG2 | Epithelium | Liver | REST | human | TF | HepG2 |
| 8 | 45802 | 1 | 50025424 | 50025836 | 22.95895 | -73 | RACGAP1 | promoter-TSS (NM_001320003) | 153.695 | 149.1766 | chr12 | GSM935307 | HepG2 | Epithelium | Liver | CHD2 | human | TF | HepG2 |
| 29 | 46234 | 1 | 50025410 | 50025849 | 9.74826 | -72 | RACGAP1 | promoter-TSS (NM_001320003) | 39.60203 | 36.51085 | chr12 | GSM803507 | HepG2 | Epithelium | Liver | SP1 | human | TF | HepG2 |
| 53 | 56043 | 1 | 50025364 | 50025893 | 20.79946 | -71 | RACGAP1 | promoter-TSS (NM_001320003) | 89.94569 | 86.00244 | chr12 | GSM1861925 | HepG2 | Epithelium | Liver | GABPA | human | TF | HepG2 cells |
| 35 | 46240 | 1 | 50025305 | 50025946 | 6.95814 | -68 | RACGAP1 | promoter-TSS (NM_001320003) | 26.60477 | 23.25516 | chr12 | GSM1010741 | HepG2 | Epithelium | Liver | NFIC | human | TF | HepG2 |
| 54 | 56044 | 1 | 50025388 | 50025863 | 28.51978 | -68 | RACGAP1 | promoter-TSS (NM_001320003) | 204.1506 | 199.8243 | chr12 | GSM1861926 | HepG2 | Epithelium | Liver | GABPA | human | TF | HepG2 cells |
| 14 | 45817 | 1 | 50025532 | 50025715 | 5.56439 | -66 | RACGAP1 | promoter-TSS (NM_001320003) | 11.54969 | 9.03295 | chr12 | GSM935610 | HepG2 | Epithelium | Liver | MAFK | human | TF | HepG2 |
| 4 | 45791 | 1 | 50025345 | 50025900 | 7.57638 | -65 | RACGAP1 | promoter-TSS (NM_001320003) | 22.97701 | 19.72146 | chr12 | GSM935280 | HepG2 | Epithelium | Liver | TBP | human | TF | HepG2 |
| 24 | 46226 | 1 | 50025261 | 50025984 | 23.07337 | -65 | RACGAP1 | promoter-TSS (NM_001320003) | 267.5005 | 262.1187 | chr12 | GSM803381 | HepG2 | Epithelium | Liver | YY1 | human | TF | HepG2 |
| 23 | 46225 | 1 | 50025482 | 50025752 | 5.52205 | -60 | RACGAP1 | promoter-TSS (NM_001320003) | 13.35353 | 10.71657 | chr12 | GSM803499 | HepG2 | Epithelium | Liver | EP300 | human | TF | HepG2 |
| 7 | 45801 | 1 | 50025477 | 50025750 | 11.59385 | -56 | RACGAP1 | promoter-TSS (NM_001320003) | 32.09743 | 29.21679 | chr12 | GSM935306 | HepG2 | Epithelium | Liver | MAFF | human | TF | HepG2 |
| 37 | 46242 | 1 | 50025390 | 50025836 | 6.63221 | -56 | RACGAP1 | promoter-TSS (NM_001320003) | 17.04869 | 14.1866 | chr12 | GSM803452 | HepG2 | Epithelium | Liver | RXRA | human | TF | HepG2 |
| 51 | 56041 | 1 | 50025412 | 50025813 | 5.0338 | -55 | RACGAP1 | promoter-TSS (NM_001320003) | 8.14957 | 5.80286 | chr12 | GSM1861923 | HepG2 | Epithelium | Liver | RAD21 | human | TF | HepG2 cells |
| 21 | 46223 | 1 | 50025394 | 50025826 | 4.70831 | -53 | RACGAP1 | promoter-TSS (NM_001320003) | 9.81628 | 6.65478 | chr12 | GSM803502 | HepG2 | Epithelium | Liver | SRF | human | TF | HepG2 |
| 26 | 46229 | 1 | 50025401 | 50025817 | 13.81979 | -52 | RACGAP1 | promoter-TSS (NM_001320003) | 66.48989 | 62.04778 | chr12 | GSM803449 | HepG2 | Epithelium | Liver | ZBTB33 | human | TF | HepG2 |
| 20 | 45828 | 1 | 50025466 | 50025747 | 8.35612 | -49 | RACGAP1 | promoter-TSS (NM_001320003) | 22.67997 | 19.79695 | chr12 | GSM935649 | HepG2 | Epithelium | Liver | JUND | human | TF | HepG2 |
| 56 | 56046 | 1 | 50025342 | 50025870 | 25.33416 | -49 | RACGAP1 | promoter-TSS (NM_001320003) | 230.8362 | 226.3033 | chr12 | GSM1861928 | HepG2 | Epithelium | Liver | GABPA | human | TF | HepG2 cells |
| 12 | 45810 | 1 | 50025289 | 50025917 | 12.93391 | -46 | RACGAP1 | promoter-TSS (NM_001320003) | 73.0923 | 69.21747 | chr12 | GSM935437 | HepG2 | Epithelium | Liver | MXI1 | human | TF | HepG2 |
| 46 | 46254 | 1 | 50025315 | 50025892 | 24.25471 | -46 | RACGAP1 | promoter-TSS (NM_001320003) | 218.567 | 213.6741 | chr12 | GSM803415 | HepG2 | Epithelium | Liver | ELF1 | human | TF | HepG2 |
| 9 | 45804 | 1 | 50025493 | 50025710 | 7.59878 | -44 | RACGAP1 | promoter-TSS (NM_001320003) | 22.53431 | 18.93289 | chr12 | GSM935646 | HepG2 | Epithelium | Liver | USF2 | human | TF | HepG2 |
| 43 | 46249 | 1 | 50025302 | 50025895 | 6.61546 | -41 | RACGAP1 | promoter-TSS (NM_001320003) | 22.37663 | 19.27826 | chr12 | GSM1010810 | HepG2 | Epithelium | Liver | NR2F2 | human | TF | HepG2 |
| 40 | 46245 | 1 | 50025243 | 50025948 | 6.92544 | -38 | RACGAP1 | promoter-TSS (NM_001320003) | 23.23601 | 19.81542 | chr12 | GSM1010809 | HepG2 | Epithelium | Liver | ZEB1 | human | TF | HepG2 |
| 59 | 56049 | 1 | 50025426 | 50025759 | 26.26894 | -35 | RACGAP1 | promoter-TSS (NM_001320003) | 135.0583 | 130.2643 | chr12 | GSM1861931 | HepG2 | Epithelium | Liver | CREB1 | human | TF | HepG2 cells |
| 28 | 46232 | 1 | 50025317 | 50025866 | 7.65038 | -34 | RACGAP1 | promoter-TSS (NM_001320003) | 20.66864 | 17.69884 | chr12 | GSM803500 | HepG2 | Epithelium | Liver | JUND | human | TF | HepG2 |
| 61 | 56051 | 1 | 50025385 | 50025796 | 27.73053 | -33 | RACGAP1 | promoter-TSS (NM_001320003) | 168.0049 | 163.4339 | chr12 | GSM1861933 | HepG2 | Epithelium | Liver | CREB1 | human | TF | HepG2 cells |
| 11 | 45809 | 1 | 50025472 | 50025705 | 6.28634 | -31 | RACGAP1 | promoter-TSS (NM_001320003) | 14.16695 | 10.11711 | chr12 | GSM935609 | HepG2 | Epithelium | Liver | BRCA1 | human | TF | HepG2 |
| 63 | 56053 | 1 | 50025369 | 50025806 | 26.78071 | -30 | RACGAP1 | promoter-TSS (NM_001320003) | 206.4839 | 201.5704 | chr12 | GSM1861935 | HepG2 | Epithelium | Liver | ATF1 | human | TF | HepG2 cells |
| 60 | 56050 | 1 | 50025393 | 50025779 | 27.7261 | -29 | RACGAP1 | promoter-TSS (NM_001320003) | 170.0247 | 165.4143 | chr12 | GSM1861932 | HepG2 | Epithelium | Liver | CREB1 | human | TF | HepG2 cells |
| 15 | 45821 | 1 | 50025240 | 50025925 | 10.86044 | -25 | RACGAP1 | promoter-TSS (NM_001320003) | 34.4014 | 30.81605 | chr12 | GSM822284 | HepG2 | Epithelium | Liver | POLR2A | human | TF | HepG2 |
| 17 | 45823 | 1 | 50025429 | 50025736 | 8.07139 | -25 | RACGAP1 | promoter-TSS (NM_001320003) | 23.62312 | 19.92358 | chr12 | GSM935304 | HepG2 | Epithelium | Liver | RFX5 | human | TF | HepG2 |
| 16 | 45822 | 1 | 50025451 | 50025708 | 4.88331 | -22 | RACGAP1 | promoter-TSS (NM_001320003) | 9.51398 | 6.38552 | chr12 | GSM935545 | HepG2 | Epithelium | Liver | EP300 | human | TF | HepG2 |
| 64 | 56054 | 1 | 50025345 | 50025807 | 24.33244 | -19 | RACGAP1 | promoter-TSS (NM_001320003) | 161.8857 | 157.1901 | chr12 | GSM1861936 | HepG2 | Epithelium | Liver | ATF1 | human | TF | HepG2 cells |
| 38 | 46243 | 1 | 50025343 | 50025800 | 8.3826 | -14 | RACGAP1 | promoter-TSS (NM_001320003) | 25.38144 | 22.42641 | chr12 | GSM803451 | HepG2 | Epithelium | Liver | FOSL2 | human | TF | HepG2 |
| 58 | 56048 | 1 | 50025350 | 50025784 | 24.06377 | -10 | RACGAP1 | promoter-TSS (NM_001320003) | 197.5592 | 192.7687 | chr12 | GSM1861930 | HepG2 | Epithelium | Liver | CREB1 | human | TF | HepG2 cells |
| 36 | 46241 | 1 | 50025237 | 50025881 | 11.85918 | -2 | RACGAP1 | promoter-TSS (NM_001320003) | 92.29012 | 87.0339 | chr12 | GSM1010876 | HepG2 | Epithelium | Liver | MYBL2 | human | TF | HepG2 |
| 62 | 56052 | 1 | 50025342 | 50025772 | 25.71208 | 0 | RACGAP1 | promoter-TSS (NM_001320003) | 191.8193 | 187.125 | chr12 | GSM1861934 | HepG2 | Epithelium | Liver | CREB1 | human | TF | HepG2 cells |
| 27 | 46231 | 1 | 50025246 | 50025867 | 20.58241 | 1 | RACGAP1 | promoter-TSS (NM_001320003) | 253.5402 | 248.166 | chr12 | GSM1010808 | HepG2 | Epithelium | Liver | CREB1 | human | TF | HepG2 |
| 50 | 56040 | 1 | 50025423 | 50025682 | 5.22867 | 5 | RACGAP1 | promoter-TSS (NM_001320003) | 8.80116 | 6.46293 | chr12 | GSM1861922 | HepG2 | Epithelium | Liver | RAD21 | human | TF | HepG2 cells |
| 19 | 45827 | 1 | 50025323 | 50025777 | 11.39022 | 7 | RACGAP1 | promoter-TSS (NM_001320003) | 31.06944 | 27.0463 | chr12 | GSM822291 | HepG2 | Epithelium | Liver | MYC | human | TF | HepG2 |
| 39 | 46244 | 1 | 50025266 | 50025833 | 11.95847 | 8 | RACGAP1 | promoter-TSS (NM_001320003) | 90.04272 | 86.39821 | chr12 | GSM1010865 | HepG2 | Epithelium | Liver | MAX | human | TF | HepG2 |
| 3 | 45790 | 1 | 50025432 | 50025664 | 5.86354 | 9 | RACGAP1 | promoter-TSS (NM_001320003) | 8.58133 | 5.81872 | chr12 | GSM935619 | HepG2 | Epithelium | Liver | HNF4A | human | TF | HepG2 |
| 18 | 45826 | 1 | 50025239 | 50025857 | 11.73745 | 9 | RACGAP1 | promoter-TSS (NM_001320003) | 57.50399 | 54.11602 | chr12 | GSM935603 | HepG2 | Epithelium | Liver | POLR2A | human | TF | HepG2 |
| 32 | 46237 | 1 | 50025256 | 50025840 | 17.8682 | 9 | RACGAP1 | promoter-TSS (NM_001320003) | 72.90479 | 69.32474 | chr12 | GSM803343 | HepG2 | Epithelium | Liver | GABPA | human | TF | HepG2 |
| 5 | 45797 | 1 | 50025381 | 50025700 | 11.20187 | 17 | RACGAP1 | promoter-TSS (NM_001320003) | 37.69677 | 33.85657 | chr12 | GSM935406 | HepG2 | Epithelium | Liver | MAX | human | TF | HepG2 |
| 57 | 56047 | 1 | 50025255 | 50025825 | 27.24376 | 17 | RACGAP1 | promoter-TSS (NM_001320003) | 265.876 | 260.7033 | chr12 | GSM1861929 | HepG2 | Epithelium | Liver | CREB1 | human | TF | HepG2 cells |
| 42 | 46248 | 1 | 50025357 | 50025702 | 17.83355 | 28 | RACGAP1 | promoter-TSS (NM_001320003) | 122.8903 | 119.0411 | chr12 | GSM803527 | HepG2 | Epithelium | Liver | USF1 | human | TF | HepG2 |
| 10 | 45806 | 1 | 50025352 | 50025697 | 4.90043 | 33 | RACGAP1 | promoter-TSS (NM_001320003) | 10.55478 | 7.82155 | chr12 | GSM935275 | HepG2 | Epithelium | Liver | ARID3A | human | TF | HepG2 |
| 49 | 46258 | 1 | 50025204 | 50025839 | 17.78531 | 36 | RACGAP1 | promoter-TSS (NM_001320003) | 80.1628 | 76.55518 | chr12 | GSM803367 | HepG2 | Epithelium | Liver | TAF1 | human | TF | HepG2 |
| 69 | 8107 | 1 | 50025361 | 50025670 | 9.17116 | 42 | RACGAP1 | promoter-TSS (NM_001320003) | 15.67383 | 11.58698 | chr12 | GSM748542 | HepG2 | Epithelium | Liver | MYC | human | TF | HepG2_cMYC |
| 31 | 46236 | 1 | 50025183 | 50025829 | 14.19181 | 51 | RACGAP1 | promoter-TSS (NM_001320003) | 78.69578 | 74.28362 | chr12 | GSM803530 | HepG2 | Epithelium | Liver | SIN3A | human | TF | HepG2 |
| 68 | 8105 | 1 | 50025323 | 50025603 | 9.82029 | 94 | RACGAP1 | promoter-TSS (NM_001320003) | 18.50216 | 14.35169 | chr12 | GSM748540 | HepG2 | Epithelium | Liver | MYC | human | TF | HepG2_cMYC |
| 30 | 46235 | 1 | 50025161 | 50025759 | 6.40135 | 97 | RACGAP1 | promoter-TSS (NM_001320003) | 19.14457 | 16.11867 | chr12 | GSM803493 | HepG2 | Epithelium | Liver | HDAC2 | human | TF | HepG2 |
