# Supplementary material for: Up-Regulation of RACGAP1 Promotes Progressions of Hepatocellular Carcinoma Regulated by GABPA via PI3K/AKT Pathway
Source: Oxid Med Cell Longev. 2022 Aug 2;2022:3034150. doi: 10.1155/2022/3034150 (PMC9363186; doi:10.1155/2022/3034150)
Supplement: Supplementary Materials — Supplementary Figure 1: Over-expression of RACGAP1 promotes HCC growth (A and B) and metastasis (C and D) in SMMC7721. ∗∗p <0.01; ∗∗∗p <0.001. Table S1: The sequences of all primers and the information of all antibodies in our study. Supplementary File: The results of GEO (File 1) and ENCODE (File 2) for transcription factors prediction of RACGAP1. [file 3034150.f1.zip › Supplementary File2.docx]

|  | sampleID | feature_type | start | end | score | dis | symbol | attri | p | q | chrom | uniqID | bs1 | bs2 | bs3 | gender | age | IP | url | species | type |
| --- | --- | --- | --- | --- | --- | --- | --- | --- | --- | --- | --- | --- | --- | --- | --- | --- | --- | --- | --- | --- | --- |
| 25 | ENCFF405THA | 1 | 50026094 | 50026578 | 16.22349 | -779 | RACGAP1 | promoter-TSS (NM_001320003) | NA | NA | chr12 | ENCSR712FAM | HepG2 | immortalized cell line | child | male | 15 year | FLAG-ZSCAN9-human | https://www.encodeproject.org/files/ENCFF405THA/@@download/ENCFF405THA.bed.gz | human | TF |
| 15 | ENCFF263AUE | 1 | 50025603 | 50026163 | 29.31266 | -326 | RACGAP1 | promoter-TSS (NM_001320003) | NA | NA | chr12 | ENCSR654ORQ | HepG2 | immortalized cell line | child | male | 15 year | FLAG-SLC30A9-human | https://www.encodeproject.org/files/ENCFF263AUE/@@download/ENCFF263AUE.bed.gz | human | TF |
| 49 | ENCFF848JMT | 1 | 50025519 | 50026209 | 24.08216 | -307 | RACGAP1 | promoter-TSS (NM_001320003) | NA | NA | chr12 | ENCSR704IGU | HepG2 | immortalized cell line | child | male | 15 year | FLAG-ZGPAT-human | https://www.encodeproject.org/files/ENCFF848JMT/@@download/ENCFF848JMT.bed.gz | human | TF |
| 38 | ENCFF655FUP | 1 | 50025671 | 50026001 | 27.97465 | -279 | RACGAP1 | promoter-TSS (NM_001320003) | NA | NA | chr12 | ENCSR826YMT | HepG2 | immortalized cell line | child | male | 15 year | FLAG-SMAD4_isoform1-human | https://www.encodeproject.org/files/ENCFF655FUP/@@download/ENCFF655FUP.bed.gz | human | TF |
| 34 | ENCFF535XNA | 1 | 50025471 | 50026155 | 3.61649 | -256 | RACGAP1 | promoter-TSS (NM_001320003) | NA | NA | chr12 | ENCSR784FYS | HepG2 | immortalized cell line | child | male | 15 year | RBFOX2-human | https://www.encodeproject.org/files/ENCFF535XNA/@@download/ENCFF535XNA.bed.gz | human | TF |
| 43 | ENCFF721SZO | 1 | 50025511 | 50026071 | 67.45839 | -234 | RACGAP1 | promoter-TSS (NM_001320003) | NA | NA | chr12 | ENCSR331ORD | HepG2 | immortalized cell line | child | male | 15 year | FLAG-CREB1-human | https://www.encodeproject.org/files/ENCFF721SZO/@@download/ENCFF721SZO.bed.gz | human | TF |
| 9 | ENCFF214TVS | 1 | 50025665 | 50025888 | 110.8696 | -219 | RACGAP1 | promoter-TSS (NM_001320003) | NA | NA | chr12 | ENCSR000BPI | HepG2 | immortalized cell line | child | male | 15 year | POLR2AphosphoS5-human | https://www.encodeproject.org/files/ENCFF214TVS/@@download/ENCFF214TVS.bed.gz | human | TF |
| 36 | ENCFF565SBG | 1 | 50025576 | 50025940 | 24.63666 | -201 | RACGAP1 | promoter-TSS (NM_001320003) | NA | NA | chr12 | ENCSR000EEL | HepG2 | immortalized cell line | child | male | 15 year | TBP-human | https://www.encodeproject.org/files/ENCFF565SBG/@@download/ENCFF565SBG.bed.gz | human | TF |
| 53 | ENCFF883BHO | 1 | 50025554 | 50025854 | 75.77556 | -147 | RACGAP1 | promoter-TSS (NM_001320003) | NA | NA | chr12 | ENCSR000BJK | HepG2 | immortalized cell line | child | male | 15 year | GABPA-human | https://www.encodeproject.org/files/ENCFF883BHO/@@download/ENCFF883BHO.bed.gz | human | TF |
| 44 | ENCFF790ENL | 1 | 50025508 | 50025878 | 46.80555 | -136 | RACGAP1 | promoter-TSS (NM_001320003) | NA | NA | chr12 | ENCSR497JLX | HepG2 | immortalized cell line | child | male | 15 year | FLAG-TEAD1-human | https://www.encodeproject.org/files/ENCFF790ENL/@@download/ENCFF790ENL.bed.gz | human | TF |
| 23 | ENCFF380IQS | 1 | 50025481 | 50025901 | 32.90449 | -134 | RACGAP1 | promoter-TSS (NM_001320003) | NA | NA | chr12 | ENCSR714YZG | HepG2 | immortalized cell line | child | male | 15 year | ETV4-human | https://www.encodeproject.org/files/ENCFF380IQS/@@download/ENCFF380IQS.bed.gz | human | TF |
| 14 | ENCFF249MMP | 1 | 50025336 | 50026020 | 45.35022 | -121 | RACGAP1 | promoter-TSS (NM_001320003) | NA | NA | chr12 | ENCSR670YPQ | HepG2 | immortalized cell line | child | male | 15 year | FLAG-DMAP1-human | https://www.encodeproject.org/files/ENCFF249MMP/@@download/ENCFF249MMP.bed.gz | human | TF |
| 3 | ENCFF120LIC | 1 | 50025475 | 50025879 | 46.0293 | -120 | RACGAP1 | promoter-TSS (NM_001320003) | NA | NA | chr12 | ENCSR117CHD | HepG2 | immortalized cell line | child | male | 15 year | FLAG-HOMEZ-human | https://www.encodeproject.org/files/ENCFF120LIC/@@download/ENCFF120LIC.bed.gz | human | TF |
| 16 | ENCFF263AUE | 1 | 50025395 | 50025955 | 41.07163 | -118 | RACGAP1 | promoter-TSS (NM_001320003) | NA | NA | chr12 | ENCSR654ORQ | HepG2 | immortalized cell line | child | male | 15 year | FLAG-SLC30A9-human | https://www.encodeproject.org/files/ENCFF263AUE/@@download/ENCFF263AUE.bed.gz | human | TF |
| 1 | ENCFF024NIL | 1 | 50025463 | 50025867 | 49.36274 | -108 | RACGAP1 | promoter-TSS (NM_001320003) | NA | NA | chr12 | ENCSR560SEP | HepG2 | immortalized cell line | child | male | 15 year | FLAG-RXRB-human | https://www.encodeproject.org/files/ENCFF024NIL/@@download/ENCFF024NIL.bed.gz | human | TF |
| 37 | ENCFF608OHW | 1 | 50025484 | 50025844 | 73.97561 | -107 | RACGAP1 | promoter-TSS (NM_001320003) | NA | NA | chr12 | ENCSR092OVN | HepG2 | immortalized cell line | child | male | 15 year | FLAG-FOXA3-human | https://www.encodeproject.org/files/ENCFF608OHW/@@download/ENCFF608OHW.bed.gz | human | TF |
| 48 | ENCFF848JMT | 1 | 50025318 | 50026008 | 91.39583 | -106 | RACGAP1 | promoter-TSS (NM_001320003) | NA | NA | chr12 | ENCSR704IGU | HepG2 | immortalized cell line | child | male | 15 year | FLAG-ZGPAT-human | https://www.encodeproject.org/files/ENCFF848JMT/@@download/ENCFF848JMT.bed.gz | human | TF |
| 33 | ENCFF503PQW | 1 | 50025590 | 50025735 | 97.91809 | -105 | RACGAP1 | promoter-TSS (NM_001320003) | NA | NA | chr12 | ENCSR770AOR | HepG2 | immortalized cell line | child | male | 15 year | FLAG-ELF3-human | https://www.encodeproject.org/files/ENCFF503PQW/@@download/ENCFF503PQW.bed.gz | human | TF |
| 56 | ENCFF926MTA | 1 | 50025449 | 50025876 | 531.7835 | -105 | RACGAP1 | promoter-TSS (NM_001320003) | NA | NA | chr12 | ENCSR269TNX | HepG2 | immortalized cell line | child | male | 15 year | FLAG-GABP-human | https://www.encodeproject.org/files/ENCFF926MTA/@@download/ENCFF926MTA.bed.gz | human | TF |
| 41 | ENCFF704DME | 1 | 50025553 | 50025763 | 187.3357 | -101 | RACGAP1 | promoter-TSS (NM_001320003) | NA | NA | chr12 | ENCSR066EBK | HepG2 | immortalized cell line | child | male | 15 year | FOXA2-human | https://www.encodeproject.org/files/ENCFF704DME/@@download/ENCFF704DME.bed.gz | human | TF |
| 19 | ENCFF295AZL | 1 | 50025488 | 50025824 | 438.9381 | -99 | RACGAP1 | promoter-TSS (NM_001320003) | NA | NA | chr12 | ENCSR101FJU | HepG2 | immortalized cell line | child | male | 15 year | ZNF384-human | https://www.encodeproject.org/files/ENCFF295AZL/@@download/ENCFF295AZL.bed.gz | human | TF |
| 50 | ENCFF862MKQ | 1 | 50025453 | 50025856 | 151.901 | -97 | RACGAP1 | promoter-TSS (NM_001320003) | NA | NA | chr12 | ENCSR954KIC | HepG2 | immortalized cell line | child | male | 15 year | FLAG-KAT8-human | https://www.encodeproject.org/files/ENCFF862MKQ/@@download/ENCFF862MKQ.bed.gz | human | TF |
| 27 | ENCFF433KGK | 1 | 50025428 | 50025864 | 29.60777 | -89 | RACGAP1 | promoter-TSS (NM_001320003) | NA | NA | chr12 | ENCSR382PVA | HepG2 | immortalized cell line | child | male | 15 year | GTF2F1-human | https://www.encodeproject.org/files/ENCFF433KGK/@@download/ENCFF433KGK.bed.gz | human | TF |
| 45 | ENCFF838QKQ | 1 | 50025501 | 50025790 | 192.0325 | -88 | RACGAP1 | promoter-TSS (NM_001320003) | NA | NA | chr12 | ENCSR529JYA | HepG2 | immortalized cell line | child | male | 15 year | HCFC1-human | https://www.encodeproject.org/files/ENCFF838QKQ/@@download/ENCFF838QKQ.bed.gz | human | TF |
| 5 | ENCFF158NBU | 1 | 50025506 | 50025775 | 162.1754 | -83 | RACGAP1 | promoter-TSS (NM_001320003) | NA | NA | chr12 | ENCSR407BEZ | HepG2 | immortalized cell line | child | male | 15 year | ZHX2-human | https://www.encodeproject.org/files/ENCFF158NBU/@@download/ENCFF158NBU.bed.gz | human | TF |
| 2 | ENCFF042DUJ | 1 | 50025427 | 50025841 | 369.8776 | -77 | RACGAP1 | promoter-TSS (NM_001320003) | NA | NA | chr12 | ENCSR000BNT | HepG2 | immortalized cell line | child | male | 15 year | YY1-human | https://www.encodeproject.org/files/ENCFF042DUJ/@@download/ENCFF042DUJ.bed.gz | human | TF |
| 30 | ENCFF482RHW | 1 | 50025422 | 50025832 | 69.46051 | -70 | RACGAP1 | promoter-TSS (NM_001320003) | NA | NA | chr12 | ENCSR993LMB | HepG2 | immortalized cell line | child | male | 15 year | FLAG-TGIF2-human | https://www.encodeproject.org/files/ENCFF482RHW/@@download/ENCFF482RHW.bed.gz | human | TF |
| 35 | ENCFF536CTX | 1 | 50025290 | 50025944 | 649.0839 | -60 | RACGAP1 | promoter-TSS (NM_001320003) | NA | NA | chr12 | ENCSR853ADA | HepG2 | immortalized cell line | child | male | 15 year | NRF1-human | https://www.encodeproject.org/files/ENCFF536CTX/@@download/ENCFF536CTX.bed.gz | human | TF |
| 47 | ENCFF846OVB | 1 | 50025400 | 50025834 | 253.5412 | -60 | RACGAP1 | promoter-TSS (NM_001320003) | NA | NA | chr12 | ENCSR562POI | HepG2 | immortalized cell line | child | male | 15 year | FLAG-THAP11-human | https://www.encodeproject.org/files/ENCFF846OVB/@@download/ENCFF846OVB.bed.gz | human | TF |
| 20 | ENCFF303TLQ | 1 | 50025365 | 50025861 | 28.63438 | -56 | RACGAP1 | promoter-TSS (NM_001320003) | NA | NA | chr12 | ENCSR372JXR | HepG2 | immortalized cell line | child | male | 15 year | FLAG-ZNF511-human | https://www.encodeproject.org/files/ENCFF303TLQ/@@download/ENCFF303TLQ.bed.gz | human | TF |
| 17 | ENCFF273IOC | 1 | 50025477 | 50025747 | 27.06414 | -55 | RACGAP1 | promoter-TSS (NM_001320003) | NA | NA | chr12 | ENCSR000EEC | HepG2 | immortalized cell line | child | male | 15 year | MAFF-human | https://www.encodeproject.org/files/ENCFF273IOC/@@download/ENCFF273IOC.bed.gz | human | TF |
| 4 | ENCFF130WFH | 1 | 50025468 | 50025728 | 337.0466 | -41 | RACGAP1 | promoter-TSS (NM_001320003) | NA | NA | chr12 | ENCSR253OON | HepG2 | immortalized cell line | child | male | 15 year | FLAG-ATF1-human | https://www.encodeproject.org/files/ENCFF130WFH/@@download/ENCFF130WFH.bed.gz | human | TF |
| 54 | ENCFF916WZZ | 1 | 50025372 | 50025823 | 56.32835 | -40 | RACGAP1 | promoter-TSS (NM_001320003) | NA | NA | chr12 | ENCSR903ELW | HepG2 | immortalized cell line | child | male | 15 year | CREM-human | https://www.encodeproject.org/files/ENCFF916WZZ/@@download/ENCFF916WZZ.bed.gz | human | TF |
| 55 | ENCFF916WZZ | 1 | 50025372 | 50025823 | 383.5853 | -40 | RACGAP1 | promoter-TSS (NM_001320003) | NA | NA | chr12 | ENCSR903ELW | HepG2 | immortalized cell line | child | male | 15 year | CREM-human | https://www.encodeproject.org/files/ENCFF916WZZ/@@download/ENCFF916WZZ.bed.gz | human | TF |
| 46 | ENCFF842LKY | 1 | 50025338 | 50025854 | 36.99253 | -39 | RACGAP1 | promoter-TSS (NM_001320003) | NA | NA | chr12 | ENCSR173CTF | HepG2 | immortalized cell line | child | male | 15 year | FLAG-ZNF580-human | https://www.encodeproject.org/files/ENCFF842LKY/@@download/ENCFF842LKY.bed.gz | human | TF |
| 42 | ENCFF721SZO | 1 | 50025430 | 50025754 | 550.9256 | -35 | RACGAP1 | promoter-TSS (NM_001320003) | NA | NA | chr12 | ENCSR331ORD | HepG2 | immortalized cell line | child | male | 15 year | FLAG-CREB1-human | https://www.encodeproject.org/files/ENCFF721SZO/@@download/ENCFF721SZO.bed.gz | human | TF |
| 18 | ENCFF284NPD | 1 | 50025404 | 50025768 | 39.53825 | -29 | RACGAP1 | promoter-TSS (NM_001320003) | NA | NA | chr12 | ENCSR125DAD | HepG2 | immortalized cell line | child | male | 15 year | FLAG-MLX-human | https://www.encodeproject.org/files/ENCFF284NPD/@@download/ENCFF284NPD.bed.gz | human | TF |
| 21 | ENCFF338JPJ | 1 | 50025387 | 50025783 | 71.11002 | -28 | RACGAP1 | promoter-TSS (NM_001320003) | NA | NA | chr12 | ENCSR966PJY | HepG2 | immortalized cell line | child | male | 15 year | FLAG-MIXL1-human | https://www.encodeproject.org/files/ENCFF338JPJ/@@download/ENCFF338JPJ.bed.gz | human | TF |
| 32 | ENCFF498PHD | 1 | 50025444 | 50025725 | 209.4589 | -27 | RACGAP1 | promoter-TSS (NM_001320003) | NA | NA | chr12 | ENCSR261EDU | HepG2 | immortalized cell line | child | male | 15 year | MNT-human | https://www.encodeproject.org/files/ENCFF498PHD/@@download/ENCFF498PHD.bed.gz | human | TF |
| 24 | ENCFF396EWL | 1 | 50025448 | 50025718 | 23.56986 | -26 | RACGAP1 | promoter-TSS (NM_001320003) | NA | NA | chr12 | ENCSR000EEI | HepG2 | immortalized cell line | child | male | 15 year | JUND-human | https://www.encodeproject.org/files/ENCFF396EWL/@@download/ENCFF396EWL.bed.gz | human | TF |
| 57 | ENCFF935AGY | 1 | 50025469 | 50025698 | 108.9202 | -26 | RACGAP1 | promoter-TSS (NM_001320003) | NA | NA | chr12 | ENCSR071YVR | HepG2 | immortalized cell line | child | male | 15 year | FLAG-KMT2B-human | https://www.encodeproject.org/files/ENCFF935AGY/@@download/ENCFF935AGY.bed.gz | human | TF |
| 29 | ENCFF469QQF | 1 | 50025433 | 50025730 | 181.6959 | -24 | RACGAP1 | promoter-TSS (NM_001320003) | NA | NA | chr12 | ENCSR108TYQ | HepG2 | immortalized cell line | child | male | 15 year | FLAG-GATAD1-human | https://www.encodeproject.org/files/ENCFF469QQF/@@download/ENCFF469QQF.bed.gz | human | TF |
| 28 | ENCFF467MXY | 1 | 50025390 | 50025760 | 84.52161 | -18 | RACGAP1 | promoter-TSS (NM_001320003) | NA | NA | chr12 | ENCSR666QNP | HepG2 | immortalized cell line | child | male | 15 year | FLAG-TEAD3-human | https://www.encodeproject.org/files/ENCFF467MXY/@@download/ENCFF467MXY.bed.gz | human | TF |
| 31 | ENCFF492YUE | 1 | 50025453 | 50025698 | 177.427 | -18 | RACGAP1 | promoter-TSS (NM_001320003) | NA | NA | chr12 | ENCSR745VSQ | HepG2 | immortalized cell line | child | male | 15 year | FLAG-GMEB2-human | https://www.encodeproject.org/files/ENCFF492YUE/@@download/ENCFF492YUE.bed.gz | human | TF |
| 11 | ENCFF236SLK | 1 | 50025258 | 50025878 | 35.17471 | -11 | RACGAP1 | promoter-TSS (NM_001320003) | NA | NA | chr12 | ENCSR616OSG | HepG2 | immortalized cell line | child | male | 15 year | FLAG-KLF11-human | https://www.encodeproject.org/files/ENCFF236SLK/@@download/ENCFF236SLK.bed.gz | human | TF |
| 12 | ENCFF238MIV | 1 | 50025399 | 50025723 | 35.51425 | -4 | RACGAP1 | promoter-TSS (NM_001320003) | NA | NA | chr12 | ENCSR000EDS | HepG2 | immortalized cell line | child | male | 15 year | MAX-human | https://www.encodeproject.org/files/ENCFF238MIV/@@download/ENCFF238MIV.bed.gz | human | TF |
| 52 | ENCFF882PRQ | 1 | 50025227 | 50025891 | 398.8622 | -2 | RACGAP1 | promoter-TSS (NM_001320003) | NA | NA | chr12 | ENCSR462KYU | HepG2 | immortalized cell line | child | male | 15 year | FLAG-GABPB1_isoform2-human | https://www.encodeproject.org/files/ENCFF882PRQ/@@download/ENCFF882PRQ.bed.gz | human | TF |
| 51 | ENCFF872LJE | 1 | 50025383 | 50025706 | 401.8405 | 13 | RACGAP1 | promoter-TSS (NM_001320003) | NA | NA | chr12 | ENCSR569ARC | HepG2 | immortalized cell line | child | male | 15 year | FLAG-NFYC-human | https://www.encodeproject.org/files/ENCFF872LJE/@@download/ENCFF872LJE.bed.gz | human | TF |
| 7 | ENCFF189NTN | 1 | 50025224 | 50025843 | 217.913 | 24 | RACGAP1 | promoter-TSS (NM_001320003) | NA | NA | chr12 | ENCSR959OMS | HepG2 | immortalized cell line | child | male | 15 year | FLAG-SAP130-human | https://www.encodeproject.org/files/ENCFF189NTN/@@download/ENCFF189NTN.bed.gz | human | TF |
| 8 | ENCFF189NTN | 1 | 50025224 | 50025843 | 99.95568 | 24 | RACGAP1 | promoter-TSS (NM_001320003) | NA | NA | chr12 | ENCSR959OMS | HepG2 | immortalized cell line | child | male | 15 year | FLAG-SAP130-human | https://www.encodeproject.org/files/ENCFF189NTN/@@download/ENCFF189NTN.bed.gz | human | TF |
| 26 | ENCFF407YHS | 1 | 50025254 | 50025804 | 28.22655 | 28 | RACGAP1 | promoter-TSS (NM_001320003) | NA | NA | chr12 | ENCSR757EKM | HepG2 | immortalized cell line | child | male | 15 year | FLAG-KLF6_isoform2-human | https://www.encodeproject.org/files/ENCFF407YHS/@@download/ENCFF407YHS.bed.gz | human | TF |
| 58 | ENCFF957NPF | 1 | 50025377 | 50025677 | 30.40697 | 30 | RACGAP1 | promoter-TSS (NM_001320003) | NA | NA | chr12 | ENCSR639IIZ | HepG2 | immortalized cell line | child | male | 15 year | FLAG-CEBPG-human | https://www.encodeproject.org/files/ENCFF957NPF/@@download/ENCFF957NPF.bed.gz | human | TF |
| 22 | ENCFF347MTY | 1 | 50025438 | 50025613 | 91.38544 | 32 | RACGAP1 | promoter-TSS (NM_001320003) | NA | NA | chr12 | ENCSR452YHM | HepG2 | immortalized cell line | child | male | 15 year | SIN3B-human | https://www.encodeproject.org/files/ENCFF347MTY/@@download/ENCFF347MTY.bed.gz | human | TF |
| 39 | ENCFF690TBO | 1 | 50025239 | 50025769 | 147.633 | 53 | RACGAP1 | promoter-TSS (NM_001320003) | NA | NA | chr12 | ENCSR988LZG | HepG2 | immortalized cell line | child | male | 15 year | FLAG-ARID4B-human | https://www.encodeproject.org/files/ENCFF690TBO/@@download/ENCFF690TBO.bed.gz | human | TF |
| 40 | ENCFF690TBO | 1 | 50025239 | 50025769 | 65.5204 | 53 | RACGAP1 | promoter-TSS (NM_001320003) | NA | NA | chr12 | ENCSR988LZG | HepG2 | immortalized cell line | child | male | 15 year | FLAG-ARID4B-human | https://www.encodeproject.org/files/ENCFF690TBO/@@download/ENCFF690TBO.bed.gz | human | TF |
| 10 | ENCFF222NHS | 1 | 50025265 | 50025701 | 67.02804 | 74 | RACGAP1 | promoter-TSS (NM_001320003) | NA | NA | chr12 | ENCSR000BGL | HepG2 | immortalized cell line | child | male | 15 year | SIN3A-human | https://www.encodeproject.org/files/ENCFF222NHS/@@download/ENCFF222NHS.bed.gz | human | TF |
| 6 | ENCFF163ZBA | 1 | 50025185 | 50025769 | 40.70128 | 80 | RACGAP1 | promoter-TSS (NM_001320003) | NA | NA | chr12 | ENCSR441KFW | HepG2 | immortalized cell line | child | male | 15 year | FLAG-MXD4-human | https://www.encodeproject.org/files/ENCFF163ZBA/@@download/ENCFF163ZBA.bed.gz | human | TF |
| 13 | ENCFF249MMP | 1 | 50025343 | 50025587 | 121.8727 | 92 | RACGAP1 | promoter-TSS (NM_001320003) | NA | NA | chr12 | ENCSR670YPQ | HepG2 | immortalized cell line | child | male | 15 year | FLAG-DMAP1-human | https://www.encodeproject.org/files/ENCFF249MMP/@@download/ENCFF249MMP.bed.gz | human | TF |
