# Supplementary material for: Up-Regulation of RACGAP1 Promotes Progressions of Hepatocellular Carcinoma Regulated by GABPA via PI3K/AKT Pathway
Source: Oxid Med Cell Longev. 2022 Aug 2;2022:3034150. doi: 10.1155/2022/3034150 (PMC9363186; doi:10.1155/2022/3034150)
Supplement: Supplementary Materials — Supplementary Figure 1: Over-expression of RACGAP1 promotes HCC growth (A and B) and metastasis (C and D) in SMMC7721. ∗∗p <0.01; ∗∗∗p <0.001. Table S1: The sequences of all primers and the information of all antibodies in our study. Supplementary File: The results of GEO (File 1) and ENCODE (File 2) for transcription factors prediction of RACGAP1. [file 3034150.f1.zip › Table S1.docx]

Primer

| Gene symbol | Sequence |
| --- | --- |
| RACGAP1-F | 5- TCCAATTTATCCAGTTGGCGAA-3 |
| RACGAP1-R | 5- CTTCAGCTTAACATCCAGAGCA-3 |
| GAPDH-F | 5- GGTATCGTGGAAGGACTCAT-3 |
| GAPDH-R | 5- CCTTGCCCACAGCCTTG-3 |
| GABPA-F | 5- AAGAACGCCTTGGGATACCCT-3 |
| GABPA-R | 5- AAGAACGCCTTGGGATACCCT-3 |

Si Sequence

| Gene sybmbol | siRNA type | Sequence |
| --- | --- | --- |
| siRACGAP1#1 | Sense | 5′-GCUGAAGCAUGCACGUAAdTdT-3 |
|  | Antisense | 5′-AUUACGUGCAUGCUUCAGdTdT-3′ |
| siRACGAP1#2 | Sense | 5′-CCCUGGACCUGUAAAGAAAdTdT-3′ |
|  | Antisense | 5′-UUUCUUUACAGGUCCAGGGdTdT-3′ |
| siRACGAP1#3 | Sense | 5′-CUCCAUUGUUGUGCAUUGUdTdT-3′ |
|  | Antisense | 5′-ACAAUGCACAACAAUGGAGdTdT-3′ |
| siGABPA#1 |  | 5-GCAGAGUGCACAGAAGAAAGCAUUG-3 |
| siGABPA#2 |  | 5-GGAGCUGAUAGAAAUUGAGAUUGAU-3 |

Antibody

| Antibody | company | type | WB | IHC/IF | IF |
| --- | --- | --- | --- | --- | --- |
| RACGAP1(ab134972) | Abcam | Rabbit monoclonal | 1:1000 | 1:200 |  |
| GABPDH(#10494-1-AP) | Proteintech | Rabbit polyclonal | 1:5000 |  |  |
| Ki67( ab92742) | Abcam | Rabbit monoclonal |  | 1:200 |  |
| Claudin1 (#13255S) | CST | Rabbit monoclonal | 1:1000 | 1:400 |  |
| Fibronectin(#26836S) | CST | Rabbit monoclonal | 1:1000 | 1:400 |  |
| Vimentin(#5741S) | CST | Rabbit monoclonal | 1:1000 | 1:400 |  |
| MMP2(#40994S) | CST | Rabbit monoclonal | 1:1000 |  |  |
| MMP9(#13667S) | CST | Rabbit monoclonal | 1:1000 |  |  |
| E-cadherin(20874-1-AP) | Proteintech | Rabbit polyclonal | 1:1000 |  |  |
| CDK2(#MA5-17052) | ThermoFisher | Mouse monoclonal | 1:1000 | 1:400 | 1:400 |
| Cyclin D1(#5555065) | CST | Rabbit monoclonal | 1:1000 |  |  |
| β-catenin(#13-8400) | ThermoFisher | Mouse monoclonal | 1:1000 | 1:400 | 1:400 |
| PI3K(#4257S) | CST | Rabbit monoclonal | 1:1000 |  |  |
| p-PI3K(#4060S) | CST | Rabbit monoclonal | 1:1000 |  |  |
| AKT(#4691S) | CST | Rabbit monoclonal | 1:1000 |  |  |
| p-AKT(#4060S) | CST | Rabbit monoclonal | 1:1000 |  |  |
| GSK3β(#12456S) | CST | Rabbit monoclonal | 1:1000 |  |  |
| p-GSK3β(#9323S) | CST | Rabbit monoclonal | 1:1000 |  |  |
| GABPA(#PA5-27735) | ThermoFisher | Rabbit polyclonal | 1:1000 |  |  |
