# Supplementary figures and images for: Up-Regulation of RACGAP1 Promotes Progressions of Hepatocellular Carcinoma Regulated by GABPA via PI3K/AKT Pathway
Source: Oxid Med Cell Longev. 2022 Aug 2;2022:3034150. doi: 10.1155/2022/3034150 (PMC9363186; doi:10.1155/2022/3034150)

**A**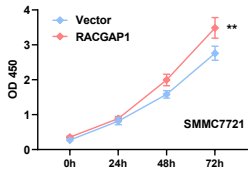**B**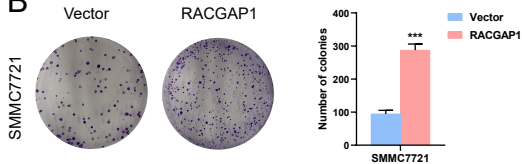**C**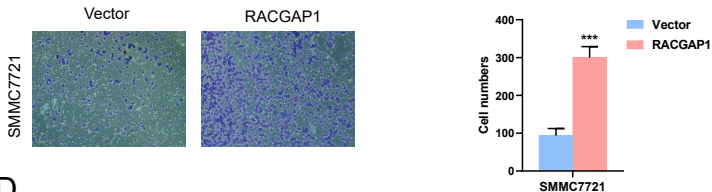**D**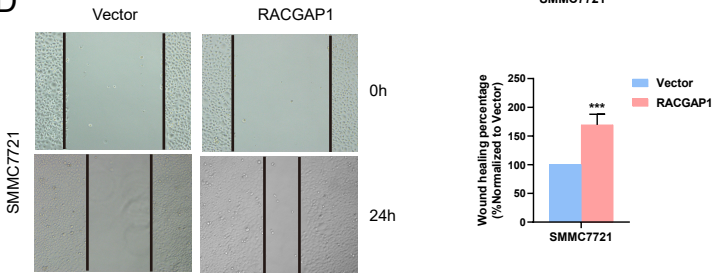

Supplement: Supplementary Materials — Supplementary Figure 1: Over-expression of RACGAP1 promotes HCC growth (A and B) and metastasis (C and D) in SMMC7721. ∗∗p <0.01; ∗∗∗p <0.001. Table S1: The sequences of all primers and the information of all antibodies in our study. Supplementary File: The results of GEO (File 1) and ENCODE (File 2) for transcription factors prediction of RACGAP1. [file 3034150.f1.zip › Supplementary Fig1 (1).pdf]
